# The mean seasonal cycle in relative sea level from satellite altimetry and gravimetry

**DOI:** 10.1007/s00190-021-01529-1

**Published:** 2021-06-27

**Authors:** Richard D. Ray, Bryant D. Loomis, Victor Zlotnicki

**Affiliations:** 1 NASA Goddard Space Flight Center, Greenbelt, MD USA; 2 Jet Propulsion Laboratory, Pasadena, CA USA

**Keywords:** Annual/semiannual cycle, Satellite altimetry, Annual land motion, Annual geocenter motion

## Abstract

Satellite altimetry and gravimetry are used to determine the mean seasonal cycle in relative sea level, a quantity relevant to coastal flooding and related applications. The main harmonics (annual, semiannual, terannual) are estimated from 25 years of gridded altimetry, while several conventional altimeter “corrections” (gravitational tide, pole tide, and inverted barometer) are restored. To transform from absolute to relative sea levels, a model of vertical land motion is developed from a high-resolution seasonal mass inversion estimated from satellite gravimetry. An adjustment for annual geocenter motion accounts for use of a center-of-mass reference frame in satellite orbit determination. A set of 544 test tide gauges, from which seasonal harmonics have been estimated from hourly measurements, is used to assess how accurately each adjustment to the altimeter data helps converge the results to true relative sea levels. At these gauges, the median annual and semiannual amplitudes are 7.1 cm and 2.2 cm, respectively. The root-mean-square differences with altimetry are 3.24 and 1.17 cm, respectively, which are reduced to 1.93 and 0.86 cm after restoration of corrections and adjustment for land motion. Example outliers highlight some limitations of present-day coastal altimetry owing to inadequate spatial resolution: upwelling and currents off Oregon and wave setup at Minamitori Island.

## Introduction

Satellite altimetry has been used to study the annual and semiannual cycles in sea level since the earliest days of altimetry (Jacobs et al. 1992; Knudsen 1994; Nerem et al. 1994). In the intervening years, studies on both regional and global scales have been conducted (e.g., Amiruddin et al. 2015; Feng et al. 2015), with at least one comprehensive study based on a large-scale numerical circulation model constrained by altimeter and other data (Vinogradov et al. 2008). Comparisons of altimeter estimates of the seasonal cycle with tide-gauge measurements have been published (Vinogradov and Ponte 2010; Ruiz Etcheverry et al. 2015), both to assess the quality or limitations of either system as well as to understand differences between strictly coastal measurements and those made in the open sea.

In nearly all previous altimeter investigations of the seasonal cycle, the goal has been, directly or indirectly, to understand ocean dynamics. Thus, two kinds of signals considered of little interest have been removed: (1) those forced by the astronomical tidal potential, which at these time scales are close to equilibrium and (2) those forced by atmospheric pressure loading, which are again close to equilibrium (inverted barometer) at these time scales (Pugh and Woodworth 2014).

In contrast, the present work is motivated by understanding the contribution of the seasonal cycle to coastal flooding and how that may be incorporated into future high-water projections, including for the many locations where altimetry may be providing the only relevant sea-level measurements. We are thus interested here in the seasonal cycle in *relative* sea level, meaning sea level relative to the adjoining land (or seafloor), equivalent to what is measured by a conventional coastal tide gauge. Moreover, we must account for all components of sea level, including those from tidal forcing, from atmospheric loading, and from some geodetic effects often overlooked. Careful examination of these various components forms the main body of this work, thus giving the paper a distinctly geodetic, as opposed to oceanographic, flavor.

In addition to altimeter data, extensive use is made here of data from 544 tide gauges, which act as “ground truth.” The data are in the form of hourly (or faster) measurements from the GESLA-2 database (Woodworth et al. 2017), from which we have estimated the seasonal components of relative sea level, specifically the amplitudes and phases of the annual, semiannual, and terannual harmonics. Details about these data are summarized in Appendix B.

The altimeter data are taken from two sources, both consisting of optimally interpolated, gridded time series. Seasonal charts from these data are the topic of Sect. 2. Both altimeter sources have used models to remove tidal and atmospheric loading signals, and these must be restored. As the altimeter-based seasonal harmonics are adjusted for these and various geodetic effects, the 544 tide-gauge data are used to confirm that the altimetry is gradually yielding, with each successive adjustment, an improved depiction of relative sea level. Sections 3–7 individually address each effect: atmospheric loading, astronomical tides, the pole tide, vertical land motion, and finally an adjustment of the altimetry for geocenter motion. Section 8 examines in some detail two problematic cases where altimetry and tide gauges diverge; each case arises ultimately from limited spatial resolution of the satellite data.

The subject here is the *mean* seasonal cycle. Yet just as one year’s weather may be more or less severe than average, the seasonal cycle in sea level also displays year-to-year variability (e.g., Feng et al. 2015). In some places interannual (decadal and longer) trends have been observed, such as off the US Gulf and east coasts (Wahl et al. 2014; Calafat et al. 2018) and in the Baltic Sea (Ekman and Stigebrandt 1990; Plag and Tsimplis 1999; Barbosa and Donner 2016). Such variability is here ignored, except that a measure of it is used to form maps of standard errors for the time-mean harmonics. Moreover, owing to this variability, the adopted tide-gauge data were selected to coincide (roughly) with the altimeter time period.

Throughout this work, the annual cycle and its first and second harmonics are generally referred to by standard tidal constituent names Sa, Ssa, and Sta (annual, semiannual, and terannual, respectively), regardless of whether the topic is the astronomical tide or the observed “meteorological tide.” This follows conventional usage. In addition, the arguments of these harmonics—and therefore the phase conventions—are taken relative to the vernal equinox, with a single exception discussed in Sect. 4. Appendix A gives further details on phase conventions, including how various inconsistencies have arisen in the published literature for the annual harmonic.

**Fig. 1:**
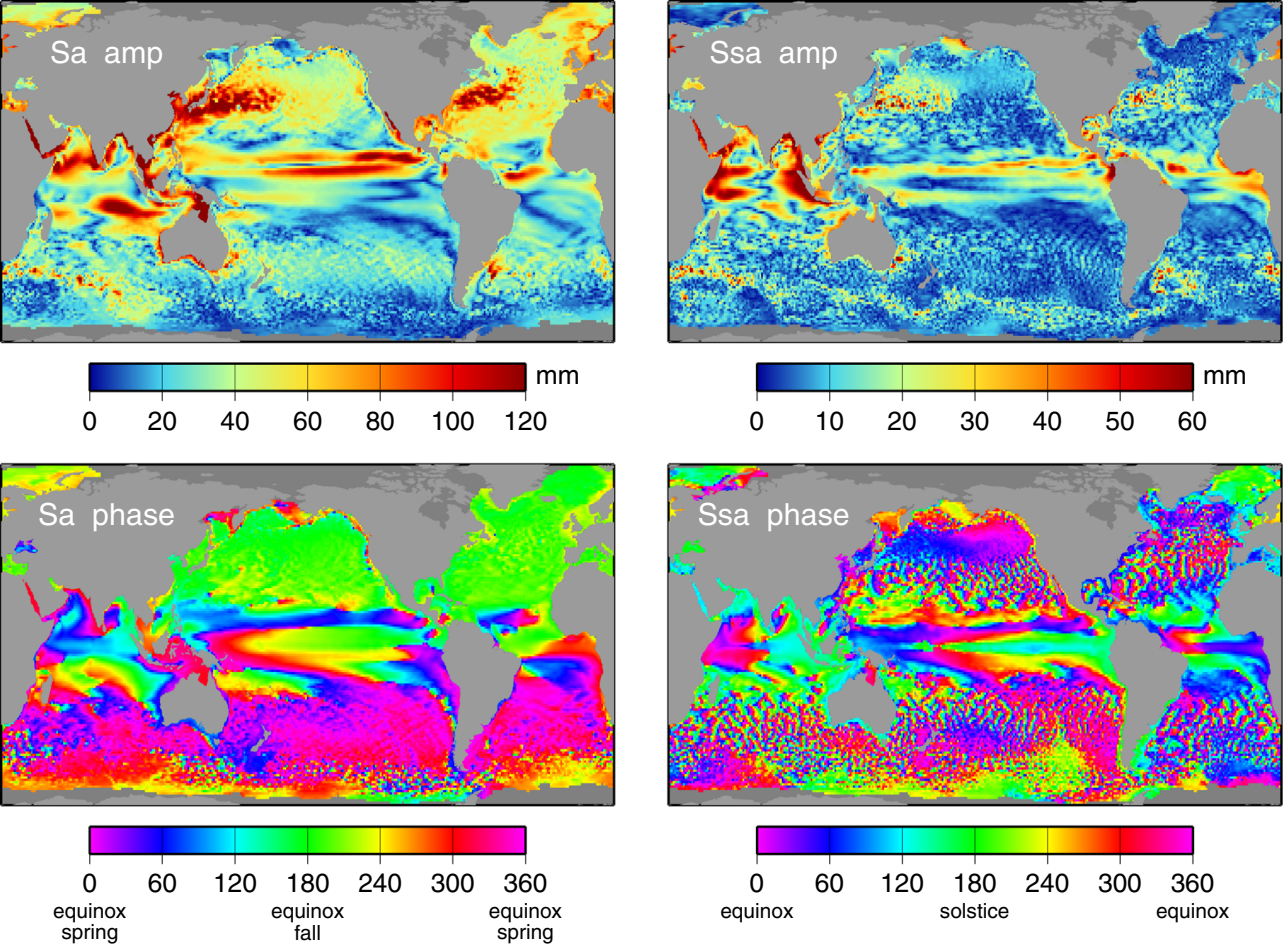
Amplitudes (top panels) and phase lags (bottom panels) of the estimated annual (Sa) and semiannual (Ssa) sea-level oscillations, deduced from DUACS gridded altimeter time series. During construction of the altimeter time series, the data were “corrected” for tides and for the inverted barometer effect, so those effects do not contribute to this figure. Phase labels of “spring” and “fall” refer to northern hemisphere seasons

## Seasonal harmonics from DUACS and MEaSUREs altimetry

The altimeter data analyzed here are from two sources, both based on gridded time series of optimally interpolated multi-satellite data. One source is the Data Unification and Altimeter Combination System (DUACS) delayed-time (DT-2018) data which are now distributed by the Copernicus Marine Service (CMEMS) and are described by Taburet et al. (2019). The DUACS time series consists of gridded sea-surface height anomalies, with spatial resolution 0.25$^{\circ }$ and 1-day sampling. The DUACS gridding algorithm used a temporal correlation scale ranging from 10 to 33 days, depending on latitude (Pujol et al. 2016), so we used data from only every fifth day. There are different varieties of DUACS solutions; we used the multi-satellite solutions which are based on consistently sampled measurements from the Topex/Poseidon, Jason-1, Jason-2, and Jason-3 missions, augmented at different times with whatever additional satellites were in operation, including those flying on nonrepeating ground-tracks.

The second source of altimetry is a product associated with the NASA project Making Earth System Data Records for Use in Research Environments (MEaSUREs). It consists of gridded sea-surface height anomalies with 5-day sampling on a (1/6)$^{\circ }$ grid. At each time step, the grids were constructed with data from two altimeters—one from the Topex/Jason series and one from either ERS, Envisat, AltiKa, CryoSat-2, or Sentinel-3A. Some further details about the generation of these data are given in Appendix C.

Aside from differences in spatial and temporal resolutions, gridding algorithms, and which satellite altimeters were used, the two altimeter products also differ in some of the adopted precise orbits and some of the fundamental corrections. The latter includes different ocean tide models; see product documentation for details.

Note that neither DUACS nor MEaSUREs data benefit from some of the recent enhancements developed for extending radar altimetry into the coastal zone, such as special retracking algorithms (Passaro et al. 2014; Vignudelli et al. 2019). Such refinements to both altimeter data products are expected in the near future, which, if successful, will result in improved relative sea levels over those we report here.

For both altimeter sources, data spanning the period 1993 through 2017 were used to estimate coefficients for the Sa, Ssa, and Sta harmonics from the time series at each individual grid point. If the posterior error covariance matrix indicated cross-correlations exceeding 0.5, the solution at that location was rejected and is excluded from the resulting maps. This proved to be a useful method for eliminating regions where excessive ice cover during too much of the year prohibited solving for an annual signal.

### Estimated harmonics

The results for the mean amplitudes and phase lags of the annual and semiannual components estimated from the DUACS data are shown in Fig. 1. The equivalent results from MEaSUREs data are nearly identical at the scale of the figure and so are not shown. These results, based on altimeter data with tides and IB removed, are similar to results previously published (e.g., Nerem et al. 1994), although here with higher spatial resolution and with somewhat less noise owing to the longer time series. As is generally known, the largest amplitudes of the annual cycle appear in the northern hemisphere off the eastern shores of large land masses. These large annual oscillations are driven primarily by surface heat fluxes (Vinogradov et al. 2008), which lead to the pronounced hemispheric phase differences in the annual cycle (Pattullo et al. 1955); Fig. 1 (lower left) shows northern hemisphere annual sea levels peak near the September equinox and southern hemisphere peak near the March equinox. Surface heating is less important to the annual cycle in lower latitudes where the wind stress and the wind stress curl are more important drivers (Gill and Niiler 1973; Vinogradov et al. 2008).

Many of the highest amplitude spots of Fig. 1 saturate the color scale, sometimes substantially so. For example, the largest amplitude for Sa is about 300 mm in the Gulf of Carpentaria. This large signal is thought to be dominated by mass flux in and out of the gulf (e.g., Vinogradov et al. 2008; Tregoning et al. 2008). The largest Sa amplitudes in the tide gauges of Appendix B occur in the Ganges River delta, but Fig. 1 shows those unusually large oscillations are confined closely to the coast.

Relatively large semiannual amplitudes occur in the northwest and northeast Indian Ocean. The very largest, exceeding 120 mm, occur in the northwest Indian, off the coast of Oman and extending westward into the Gulf of Aden. These North Indian Ocean signals have phases around 150$^{\circ }$, thus peaking several weeks before the solstices. However, the large anomalies just to the south (off the coast of Somalia) reveal phases close to 0$^{\circ }$, thus peaking near the equinoxes.

**Fig. 2:**
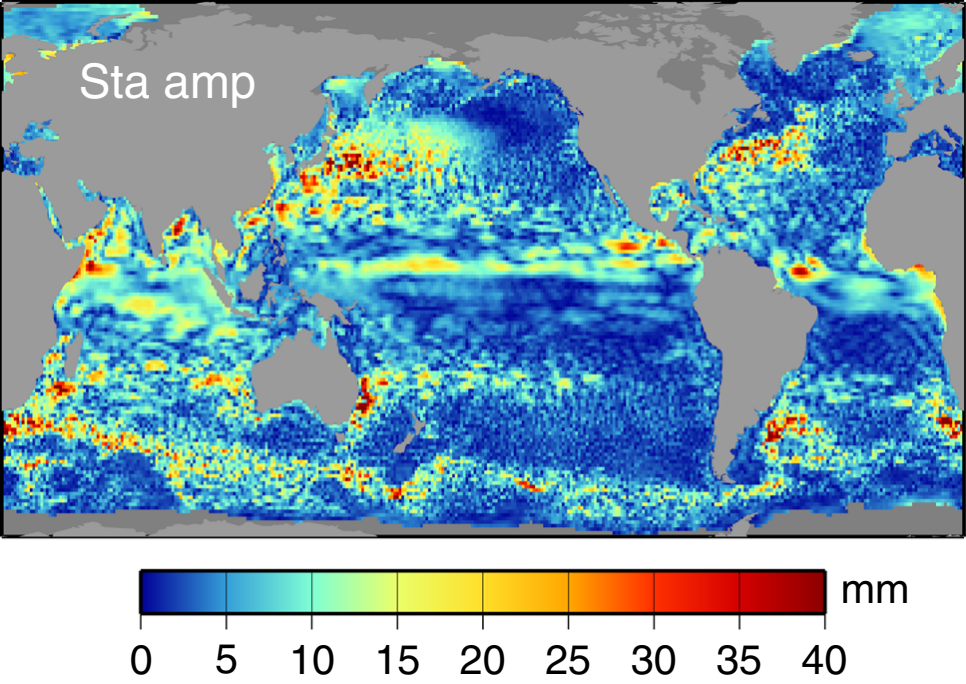
Amplitudes of the terannual Sta component of sea level, deduced as in Fig. 1 from DUACS gridded altimeter time series

The amplitudes of the terannual Sta (Fig. 2) are much smaller than those of Sa and Ssa, and the phases (not shown) are necessarily more erratic. Much of the Sta map has a distinctly noise-like appearance, with many of the highest amplitudes corresponding to locations of high mesoscale variability. This suggests that many of these signals represent merely broadband energy and are not truly a seasonal harmonic. (The same point applies to a few high-eddy locations in Sa and Ssa as well.) Estimated standard errors for these fields, discussed presently, tend to confirm this, with some notable exceptions, such as the Pacific equatorial bands. Some near-coastal enhancements of Sta are legitimately part of the seasonal cycle; Appendix B considers a case in eastern Florida. Nonetheless, because of the generally small amplitudes, we devote less attention to Sta in the remainder of this paper.

**Fig. 3:**
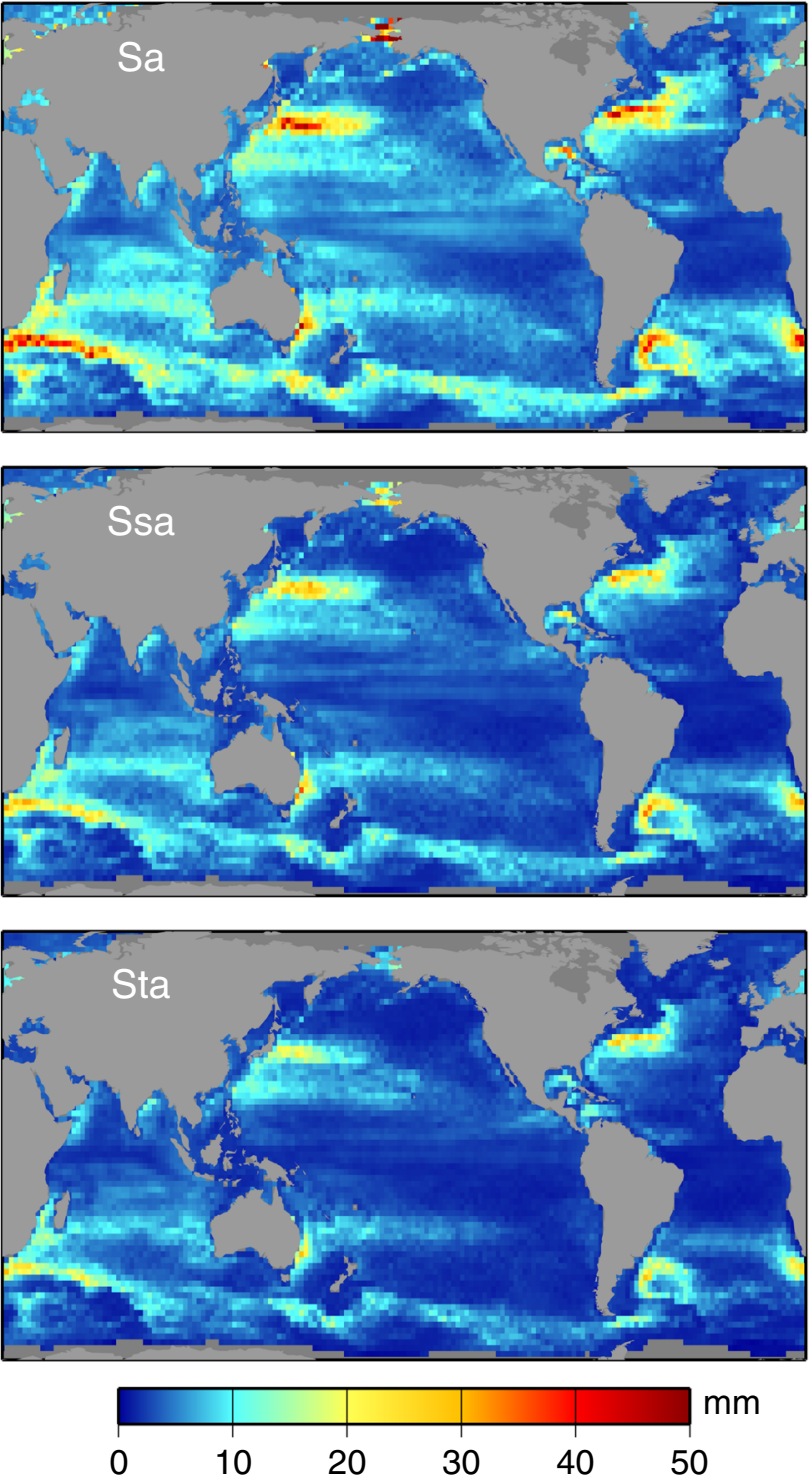
Standard errors for the three components of the seasonal sea-level cycle, as depicted in Figs. 1 and 2

Several approaches could be taken to estimate standard errors for the seasonal harmonics of Figs. 1 and 2. Any method must account for the red character of the sea level spectra across these frequencies. We have chosen an approach based on segmenting the altimeter time series into 25 yearly segments and computing Sa, Ssa, and Sta from each segment. The standard errors for Figs. 1 and 2 are then taken as the standard deviation of the yearly estimates, scaled by $1/\surd 25$. Forming instead biennial segments and scaling the standard deviations by $1/\surd 12$ gives very similar results. The results are shown in Fig. 3. These represent the standard errors $\sigma$ in the in-phase and quadrature components for each harmonic. Corresponding standard errors in amplitude *A* and phase *G* are $\sigma$ and $\sigma /A$, respectively, assuming $\sigma \ll A$; otherwise, errors in amplitude and phase are more complicated functions of $\sigma$ (Munk and Cartwright 1966, Appendix B). Reflecting in part the red sea-level spectrum, Fig. 3 shows errors are largest for Sa, smallest for Sta. It also confirms that the amplitudes of Sta are rarely much larger than the Sta estimation errors, aside from the tropical Pacific. In contrast, the annual amplitudes are generally much larger than the estimation errors.

**Table 1:** RMS differences (cm) between altimeter and tide-gauge **annual** harmonics

	MEaSUREs				DUACS			
	All	No. Am.	Europe	Japan	All	No. Am.	Europe	Japan
No. stations	544	110	163	57	544	110	163	57
RMS signal	6.639	6.139	5.756	9.439	6.639	6.139	5.756	9.439
Altimetry	3.613	4.475	2.680	4.771	3.244	3.369	2.188	4.852
+ IB	2.712	3.731	2.324	1.939	1.963	2.418	1.792	1.241
+ Sa tide	2.706	3.724	2.299	1.935	–	–	–	–
+ poletide	2.703	3.703	2.295	1.940	1.954	2.403	1.792	1.177
− VLM	2.657	3.663	2.286	1.923	1.931	2.398	1.796	1.191
+ geocenter	2.606	3.600	2.175	1.899

**Table 2:** RMS differences (cm) between altimeter and tide-gauge **semiannual** harmonics

	MEaSUREs				DUACS			
	All	No. Am.	Europe	Japan	All	No. Am.	Europe	Japan
No. stations	544	110	163	57	544	110	163	57
RMS signal	2.183	2.713	2.217	1.350	2.183	2.713	2.217	1.350
Altimetry	1.443	1.894	1.417	1.300	1.169	1.304	1.243	1.094
+ IB	1.223	1.716	1.050	0.972	0.922	1.122	0.917	0.666
+ Ssa tide	1.167	1.651	0.977	0.969	0.865	1.085	0.883	0.659
+ poletide	1.166	1.651	0.972	0.968	0.863	1.086	0.880	0.657
− VLM	1.156	1.637	0.963	0.962	0.855	1.070	0.875	0.651

### Comparison with tide gauges

Tables 1 and 2 compare the altimeter-based estimates with corresponding estimates at 544 tide gauges, for the annual and semiannual harmonics, respectively. Values from Fig. 1 have been evaluated at the tide-gauge locations by bilinear interpolation; in a few cases, extrapolation was required. The root-mean-square (RMS) differences of altimetry-minus-gauge are tabulated in the row labeled “Altimetry,” for all gauges combined and also for three dense clusters of stations from North America, Europe, and Japan (see map in Fig. 11). The RMS differences are roughly half as large as the full RMS signal from the tide gauges, indicating that the altimetry depicted in Fig. 1 captures a significant fraction of the tide-gauge variance, but by no means all of it. Since the tide-gauge data have not been corrected for tides or atmospheric loading, as the altimetry has, a great part of the residual RMS owes to this.

For all station clusters except the Japan cluster for Sa, the DUACS data are closer to the tide-gauge estimates than are the MEaSUREs data. We suspect there are several explanations for this, all related to better DUACS quality in the near-coastal zones where the majority of tide gauges are located. Possible explanations are: (a) The DUACS grids are built with data from more satellites, which likely helps capture more high-wavenumber structure typically encountered in coastal regions. (b) The DUACS interpolation algorithm has been specially tuned (relative to earlier versions such as DT-2014) to improve mapping in shallow regions (Taburet et al. 2019). (c) The FES2014 tide model (Lyard et al. 2021) used in DUACS data processing is known to be currently the most accurate global model in the near-coastal zone.

The following three sections restore to the altimetry the tidal and inverted barometer components that were removed in the DUACS and MEaSUREs data processing. Subsequent sections then adjust the data for additional geodetic effects. After each step, the RMS differences with tide gauges are compiled and listed as additional rows of Tables 1 and 2.

## Inverted barometer

Both altimeter products, DUACS and MEaSUREs, removed a dynamic atmospheric correction (DAC) based on modeling work by Carrère and Lyard (2003), with subsequent refinements. The justification for this adjustment to the altimeter data is twofold: (1) It reduces aliasing of high-frequency ocean variability by removing dynamic wind and pressure-driven variability at periods less than 20 days. A period of 20 d corresponds to the Nyquist period of the original Topex repeat sampling; the Nyquist sampling periods for other missions like Envisat differ, but the 20-d cutoff has been maintained for uniformity across missions. (2) It removes the dynamically less interesting, isostatic, pressure-driven variability at periods longer than 20 days. At the annual, semiannual, and terannual periods of interest here, it is only the second component, which is an inverted barometer (IB) response, that need be restored.

Over the years, the atmospheric pressure data used for modeling the IB effect in altimeter data have been based on different atmospheric operational and/or reanalysis products from meteorological centers. According to Taburet et al. (2019), the pressure data used in DUACS DT-2018 were generally based on the European Centre for Medium-range Weather Forecasting (ECMWF) operational data version 3.2.0, but their ERS-1 and ERS-2 satellite data employed ECMWF ERA-Interim reanalysis pressure data, as described in detail by Carrère et al. (2016). The MEaSUREs data are based mostly on ECMWF operational pressures, although the correction for Jason-1 used ERA-Interim.

We cannot restore a correction at this stage based on a mixture of different pressure products; we must use one consistent product. Ideally, this should be consistent with the pressure used for the majority of the altimeter data. We have not attempted that, but instead we employed ECMWF pressures from their latest ERA5 reanalysis (Hersbach et al. 2020). The ERA5 pressures will eventually appear in reprocessed altimeter databases, since updated corrections are currently being computed using ERA5 forcing (Loren Carrère, pers. commun., Sept 2020).

**Fig. 4:**
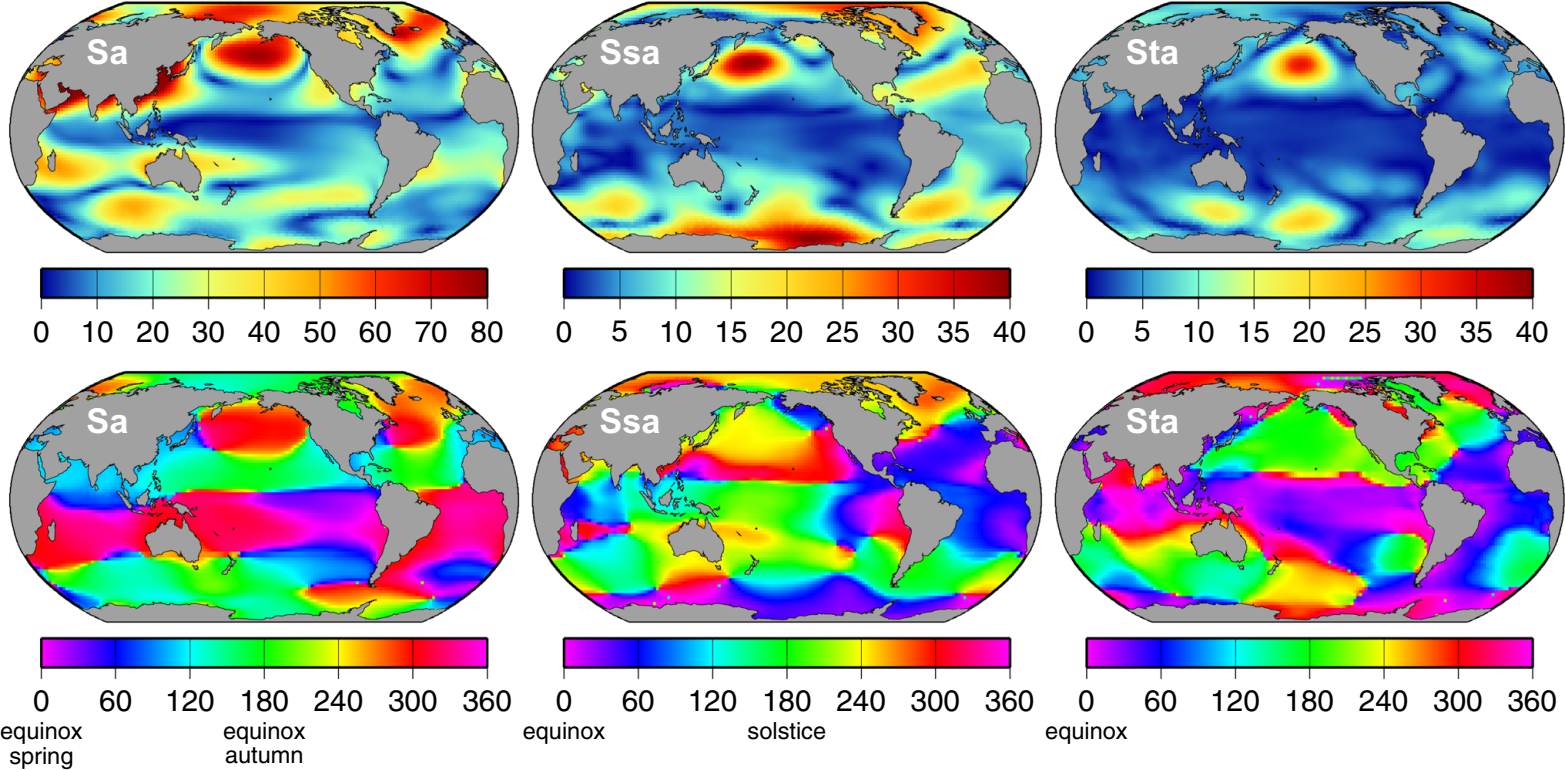
Amplitudes (top panels, in mm) and phase lags (bottom panels) of the annual (Sa), semiannual (Ssa), and terannual (Sta) components of the ocean’s inverted barometer response to loading by atmospheric surface pressures. Pressure data are from ECMWF ERA5

We have computed seasonal harmonics of the IB from ERA5 (mean sea level) pressure data for the period 1993–2017. The calculation accounts for variations in the mean pressure over the global ocean, which is predominantly an annual variation of mean amplitude approximately 0.6 hPa, maximum in June and minimum in December; the mean amplitude of a second harmonic (affecting Ssa) is only 0.08 hPa. The results for the annual, semiannual, and terannual IB terms are shown in Fig. 4. Similar color figures of annual and semiannual atmospheric sea-level pressure were published by Chen et al. (2012); the phases here differ by 180$^{\circ }$ since we are depicting the IB response to air pressure. Figure 4 shows annual variability is greatest in the northern hemisphere; it is much smaller in the southern hemisphere and is minimal along the equator. The semiannual term dominates variability in the Southern Ocean, an intriguing feature apparently induced by the different annual cycles in surface temperature between the Antarctic continent and the surrounding mid-latitude ocean (van Loon 1967; Meehl 1991; Walland and Simmonds 1999). The terannual term, so small in other quantities throughout this paper, reaches a relatively large 3.3 cm in the North Pacific, but it is rarely even 1 cm along any coastline.

Tables 1 and 2 give the RMS differences with tide gauges once the IB fields of Fig. 4 are restored to the altimetry. In all cases, the reduction in RMS relative to the original fields is pronounced. The largest relative reduction is in Sa for the stations near Japan. This is consistent with Fig. 4, which shows that the annual IB effect is large along the coasts of eastern Asia, including for much of Japan, thus pointing to the critical importance of pressure-driven annual variability to observed sea levels in that region (cf., Amiruddin et al. 2015). The phase lags in that region are around 120$^{\circ }$, indicating highest pressure-driven sea levels in mid-summer.

## Astronomical tides

All long-period tidal constituents, be they lunar or solar, are in essence modulations of the ocean’s permanent tide caused by various motions of the moon and sun. For the annual Sa tide, the modulation is induced by the annually varying distance between earth and sun. Because the eccentricity of the earth’s orbit is fairly small, the Sa astronomical potential is also fairly small. For the semiannual Ssa tide, the modulation is induced by the declinational motion of the sun, specifically its twice yearly movement away from the equator. This is a significantly larger effect than the eccentricity effect, so the Ssa tide is significantly larger than Sa. There is also a terannual term (Sta) in the potential, but it is only about a third as large as the already very small Sa (Cartwright and Tayler 1971); it is not considered further below.

**Fig. 5:**
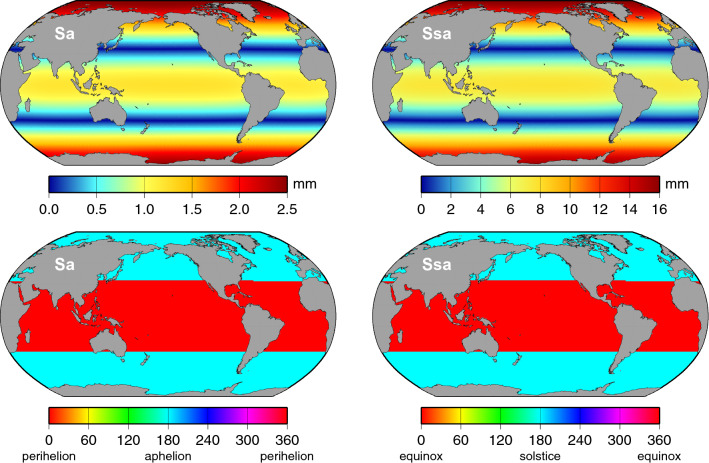
Amplitudes (top panels) and phase lags (bottom panels) of the Sa and Ssa tidal constituents, modeled as self-consistent equilibrium responses to the gravitational tidal potential. Sa is maximum along the equator when the sun is at perihelion; Ssa is maximum along the equator at spring or fall equinox when the sun is directly overhead

The largest long-period tide is the lunar fortnightly Mf constituent. Unlike the solar Sa and Ssa, which have never been unambiguously observed because of the dominating meteorological forcing of the ocean at those periods, Mf has been carefully studied and is known to be fairly close to equilibrium, although with noticeable low-latitude phase and amplitude differences between ocean basins (Egbert and Ray 2003). The longer period solar constituents should be even closer to equilibrium (Carton 1983; Ponte et al. 2015). A self-consistent equilibrium model (Agnew and Farrell 1978) in fact was used in the MEaSUREs processing (at least for Sa, Ssa and Sta; a dynamic model was used for monthly to weekly tides). The annual and semiannual amplitudes and phases (the latter being identically either 0$^{\circ }$or 180$^{\circ }$) are shown in Fig. 5, and these are the tidal fields we restored to the MEaSUREs product.

The DUACS version DT-2018 altimetry was processed with tide corrections from the FES2014 tide model of Lyard et al. (2021). Their semiannual Ssa is very close to the equilibrium model shown in Fig. 5. However, we have been unable to establish exactly which model, if any, was used for the annual constituent. The FES2014 atlas does include an Sa constituent, but it is not an equilibrium (or near-equilibrium) tide as in Fig. 5. Instead, it is a compound tide, induced evidently by nonlinear interactions between various diurnal or semidiurnal constituents separated in frequency by 1 cycle/year (e.g., by interactions between S$_2$ and T$_2$). This compound tide (not shown) is very small, less than 0.2 mm throughout most of the deep oceans, but it reaches a few mm in some shallow-water areas where nonlinear interactions are likely to occur (e.g., Gulf of Carpentaria and surrounding water). It is not clear, however, if this constituent was included in the DUACS tide corrections, because different versions of the FES2014 prediction software have and have not included Sa. Nevertheless, it is so tiny it hardly matters, so we treat the DUACS data as having no Sa tide correction.

Tables 1 and 2 give the RMS differences with tide gauges once the IB and astronomical tides have been restored (rows labeled “+ ... tides”). Because Ssa is so much larger than Sa, the reductions in RMS are clearest for Ssa. Yet the reductions are consistently positive even for Sa. The Japan stations show only a small effect from the tide corrections, which is understandable since the long-period tides have a nodal line at latitude 35$^{\circ }$, and thus very small amplitudes near Japan (Fig. 5).

## Pole tide

Altimeter data are routinely corrected for the ocean pole tide, and both DUACS and MEaSUREs projects have followed the proposed approach of Desai et al. (2015) for computing pole tides. Dr. Desai has kindly distributed software to implement that approach, which we have used here along with daily pole positions distributed by the International Earth Rotation and Reference Systems Service.

**Fig. 6:**
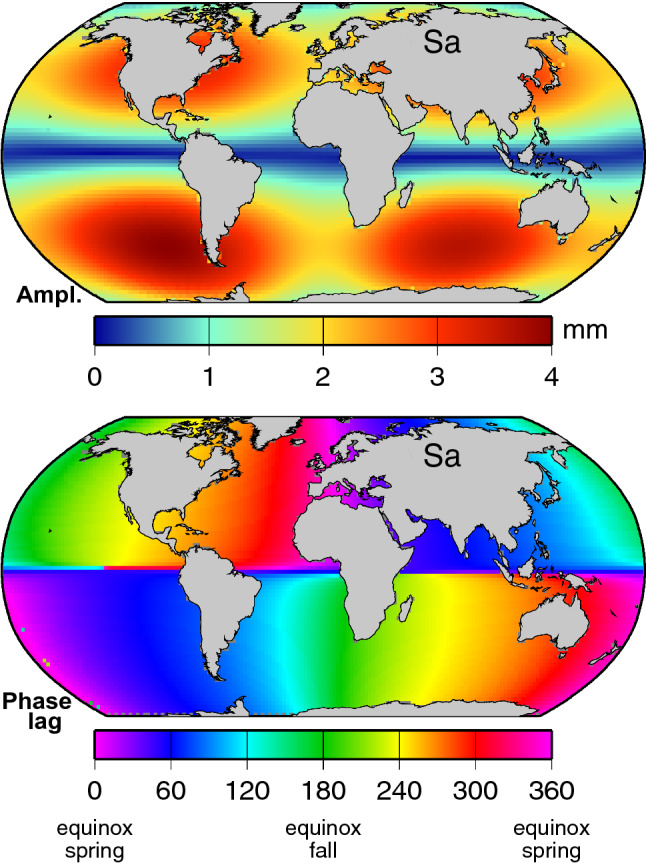
Amplitudes (top) and phase lags (bottom) of the annual pole tide (Desai et al. 2015)

We have evaluated the pole tide over the global ocean for the time span 1993–2017 and have estimated at every location the seasonal harmonics. The annual terms are shown in Fig. 6. As is well known (Lambeck 1980), the pole tide is dominated by two terms: the Chandler Wobble (period near 14 months) and the annual cycle. In fact, a semiannual term is not zero, but it is very small: over the whole global ocean, the largest semiannual amplitude is only 0.6 mm, so it could be justifiably ignored.

Tables 1 and 2 again give RMS differences once the pole tide corrections have been restored to the altimetry. All table entries indicate reductions in RMS, with two exceptions: Sa for MEaSUREs is slightly inflated for the Japan stations, and Ssa for DUACS is essentially unchanged for North American stations.

## Vertical land motion

Sections 3–5 discussed straightforward restorations of terms previously removed from the altimeter data. The present section addresses a necessary adjustment that is less straightforward: accounting for land motion to convert the altimetric sea levels to truly relative measurements.

Geodetic measurements of land motion, made for example with GPS receivers, have revealed significant annual oscillations in crustal motion (Dong et al. 2002), but the densities of space-geodetic stations are too sparse to provide a useful adjustment to altimetry except perhaps in a few highly instrumented locations such as Japan (Sagiya et al. 2000) or the contiguous United States (Lau et al. 2020), and even then interferometric synthetic aperture radar (InSAR) measurements have revealed finer-scale deformation (e.g., Buzzanga et al. 2020; Blackwell et al. 2020). Thus, given the limitations of present-day observational systems, no approach to developing a global land-motion model will be completely satisfactory. We have attempted to develop a model based on time-varying gravity measurements from the GRACE satellite mission. The methodology for deriving seasonal vertical crustal displacements from global gravity measurements is well understood and has been employed throughout the GRACE era (e.g., Davis et al. 2004; Kusche and Schrama 2005), with several studies devoted to comparing GRACE and GPS station estimates of the annual cycle (van Dam et al. 2007; Tesmer et al. 2011; Li et al. 2016; Chanard et al. 2018b). A limitation of the method is the relatively coarse spatial resolution of satellite gravity measurements, which will never match the resolution of more direct geometrical geodetic methods. But gravimetry data are available globally, and methods to refine their spatial resolution continue to improve.

The semiannual component of vertical deformation has received far less attention than the annual, presumably because it is small. Li et al. (2016) computed a stacked spectrum of crustal deformation from several hundred GPS time series, and they did observe a clear semiannual spectral peak in the vertical component, but it was approximately an order of magnitude smaller than their annual peak. Nonetheless, we include the semiannual component here, as it involves little additional effort.

Our GRACE solutions are based on the recent work by Loomis et al. (2019) which aims to maximize the spatial resolution of the recovered gravity fields for certain temporal components. Typically, gravity maps of annual variability are determined by fitting a regression model to a time series of monthly gravity fields, the latter expressed in terms of either mascons or spherical harmonics. Loomis et al., however, showed significant improvement in signal recovery and spatial resolution by instead fitting a regression model for each mascon directly from more than a decade of the Level-1B inter-satellite ranging measurements. While the focus of their work had been the estimation of mass trends, here we apply the same approach to determine global mascon grids of the annual and semiannual signals. We used GRACE ranging data from January 2003 through July 2016 to solve simultaneously for six parameters (bias, trend, annual cosine and sine, semiannual cosine and sine) for each of 41,168 mascons, where each mascon is of size 1 arc-degree. In order to capture the total gravity signal, we restored the annual and semiannual components of the atmosphere and ocean de-aliasing model that was applied when processing the Level-1B data. As is done for conventional monthly GRACE gravity products, we included additional degree-1 terms (Swenson et al., 2008), which places the solution in a reference frame whose coordinate origin is defined by the center of mass of the solid Earth (CE). Note that for the annually varying elastic deformations considered here, the CE frame is very close to a center-of-figure (CF) frame (Wu et al. 2012), which is of relevance below.

With time-varying geopotential Stokes coefficients $C_{nm}, S_{nm}$ computed to maximum degree $N=180$ from the mascon solutions, the elastic vertical deformation of the earth’s surface can be computed in the usual way (e.g., Kusche and Schrama 2005):

1 $$\begin{aligned} s_r(\theta ,\phi ,t)= & {} R \sum _{n=1}^N \sum _{m=0}^n \tilde{P}_n^m(\cos \theta ) \left\{ C_{nm}(t) \cos m\phi \right. \nonumber \\&\qquad + \left. S_{nm}(t) \sin m\phi \right\} \frac{h_n'}{1 + k_n'} \end{aligned}$$

where *R* is the earth’s radius, $\tilde{P}_n^m(\mu )$ are associated Legendre functions, and $h_n'$ and $k_n'$ are displacement and potential loading numbers as taken from Farrell (1972). Evidence is beginning to emerge for the existence of anelastic effects in seasonal deformation (Chanard et al. 2018a), which would necessitate use of complex Love numbers, but the anelastic effects are relatively small, especially in the vertical component of deformation, which is our only interest here.

**Fig. 7:**
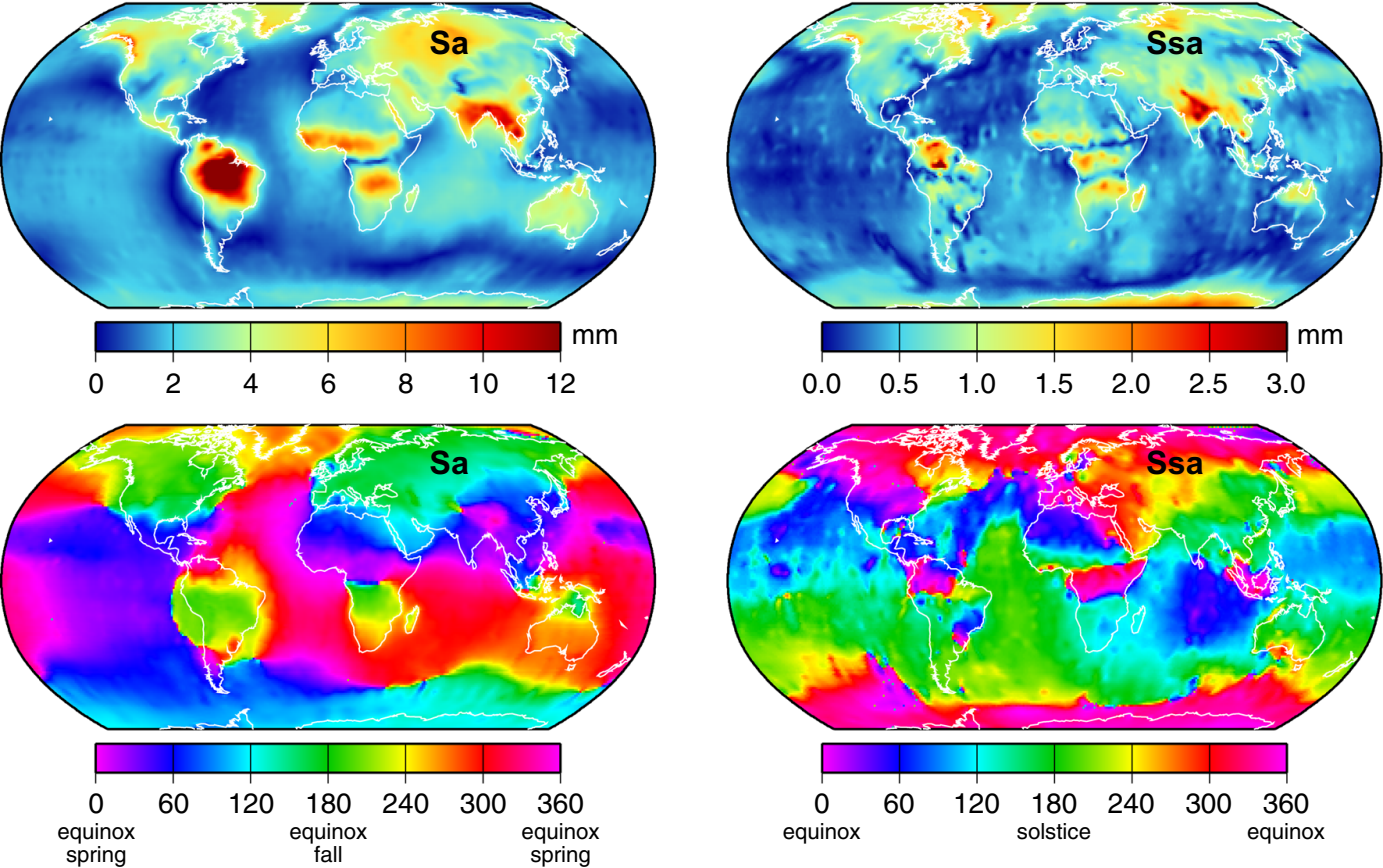
Amplitudes (top) and phase lags (bottom) of annual and semiannual components of vertical land motion, based on a high-resolution solution for annually and semiannually varying anomalous mass from GRACE range-rate data. Note different amplitude scales for annual versus semiannual

The amplitudes and phase lags of the computed vertical deformations are shown in Fig. 7. Qualitatively, the annual compares reasonably well with similar charts given by Tesmer et al. (2011) and Chanard et al. (2018b, their Fig. S10). The largest annual deformations are clearly associated with large hydrological loading over the Amazon Basin. Over the deep oceans far from land, the largest deformations occur in the central Indian Ocean. Deformations grow larger near coastlines, where the limited spatial resolution of GRACE is of most concern for comparison with tide gauges and where the relatively high resolutions of our mascon solutions will be most helpful.

Subtracting the deformation fields of Fig. 7 from the previously adjusted altimeter fields yields the sought-after relative sea levels. The comparisons with tide gauges are again given in Tables 1 and 2 (line labeled “VLM”). For most comparisons, the RMS differences show further improvement, which is a reassuring result. The only exceptions are the annual Europe and Japan clusters for DUACS data. We expect little impact on the semiannual RMS differences because the semiannual deformations are so small; nevertheless, the RMS differences are reduced in every column of Table 2.

## Geocenter

A final and more subtle adjustment arises from differences in the terrestrial reference frame used for satellite altimeter measurements and the frame needed for coastal relative sea levels. The altimeter sea-surface heights are in a reference frame that is determined solely by the satellite orbit determination. These have evolved over the years as international conventions have been updated, as algorithms have improved, and as new tracking data types became available (especially GPS). In particular, the handling of geocenter motion during orbit determination has evolved.

In the geodetic altimeter community, it is now generally the goal to place the computed satellite orbits in a center-of-mass (CM) frame and to employ processing standards that are consistent with that goal. Desai et al. (2014) discuss the issues for tidal geocenter motion. Some orbit solutions are more successful than others in reaching this goal, and approaches are still being refined. While satellites orbit the CM, the satellite tracking measurements potentially nudge the computed orbit away from this system unless the data are handled consistently. For at least a decade, geocenter corrections have been applied to satellite tracking stations to move them into a CM frame, but only for short-period tidal motions and no other motions (Lemoine et al. 2010). Even 5 years ago similar geocenter corrections for annual motion, which are of most relevance here, were not being applied owing to lack of a consensus model (Couhert et al. 2015). But that is now no longer the case, and the most recent standards (so-called GDR-E standards) include an annual adjustment to tracking stations.

The altimeter data used to generate the gridded MEaSUREs altimetric time series include data from T/P and the three Jason missions, with the orbits consistently processed following Lemoine et al. (2010) (for reference, these orbits have been labeled “std1504_dpod4”). The annual geocenter model used is based on unpublished work by J. Ries; the coefficients (N. Zelensky, pers. commun., 2020) are given in Table 3. These coefficients are very close to those of the 2014 International Terrestrial Reference Frame (Altamimi et al. 2016), and they nominally describe the motion of the CM relative to the earth’s center-of-figure (CF). (Note, however, that because the coefficients are based solely on satellite laser ranging observations, the motion is strictly relative to the center-of-network (CN) of the international laser ranging network (Collilieux et al. 2009), and this necessarily moves slightly over time as different stations come in and out of the network.)

**Table 3:** Annual geocenter motion coefficients for GSFC T/P-Jason orbits$^\mathrm{a}$

	Amp (mm)	Phase$^\mathrm{b}$
*X*	2.7	321$^{\circ }$
*Y*	2.8	241$^{\circ }$
*Z*	5.5	307$^{\circ }$

$^\mathrm{a}$ Coefficients from unpublished work of John Ries, which are close to those of Altamimi et al. (2016). Describes motion of CM relative to CF, where CF is approximated by CN of the international satellite laser ranging network.

$^\mathrm{b}$ Relative to vernal equinox

The other (non-T/P-Jason) altimeter data used in MEaSUREs processing are based on inconsistently processed orbits, some with and some without geocenter adjustments. For example, the ERS-1 and ERS-2 orbits are from Rudenko et al. (2012), and these pre-date the acceptance of the geocenter model. Because T/P-Jason were most heavily weighted in the MEaSUREs gridding algorithm, we will proceed under the assumption that the data are biased toward a CM frame, at least at the annual period.

The use of a CM frame for sea-surface heights is not widely appreciated by the oceanographic community. For most applications, it hardly matters. Nonetheless, the in situ data collected by oceanographers and the dynamics employed in ocean circulation models are not consistent with a CM frame. The tide-gauge data used here, to the extent that they are tied to a reference frame at all (some stations are leveled into national geodetic networks), are also not in a CM frame. Because the tide gauges measure water level relative to the earth’s crust, they are probably best described as being in a center-of-figure reference frame.

Thus, to compare altimetry with tide gauges requires a final adjustment of the altimetry for the CM−CF annual motion. We used the model listed in Table 3 to be consistent with that used for the T/P-Jason orbits. A radial adjustment, computed from the tabulated geocentric Cartesian coordinates, was applied. The effect of this adjustment on the RMS differences between altimetry and tide gauges is given in the final row of Table 1. There is a consistent reduction in the RMS throughout all four columns of the table. The annual geocenter adjustment has thus made the altimetry and tide-gauge data more consistent.

We are not in a position to determine what reference frame best describes the DUACS data. As with MEaSUREs data, different frames have been used for different altimeter satellites, and how these were weighted is unknown. We can report that attempts to apply the same geocenter adjustment as used for MEaSUREs did not result in improved RMS agreement with the tide-gauge data. So the final row in Table 1 for DUACS is left blank.

## Discussion

**Fig. 8:**
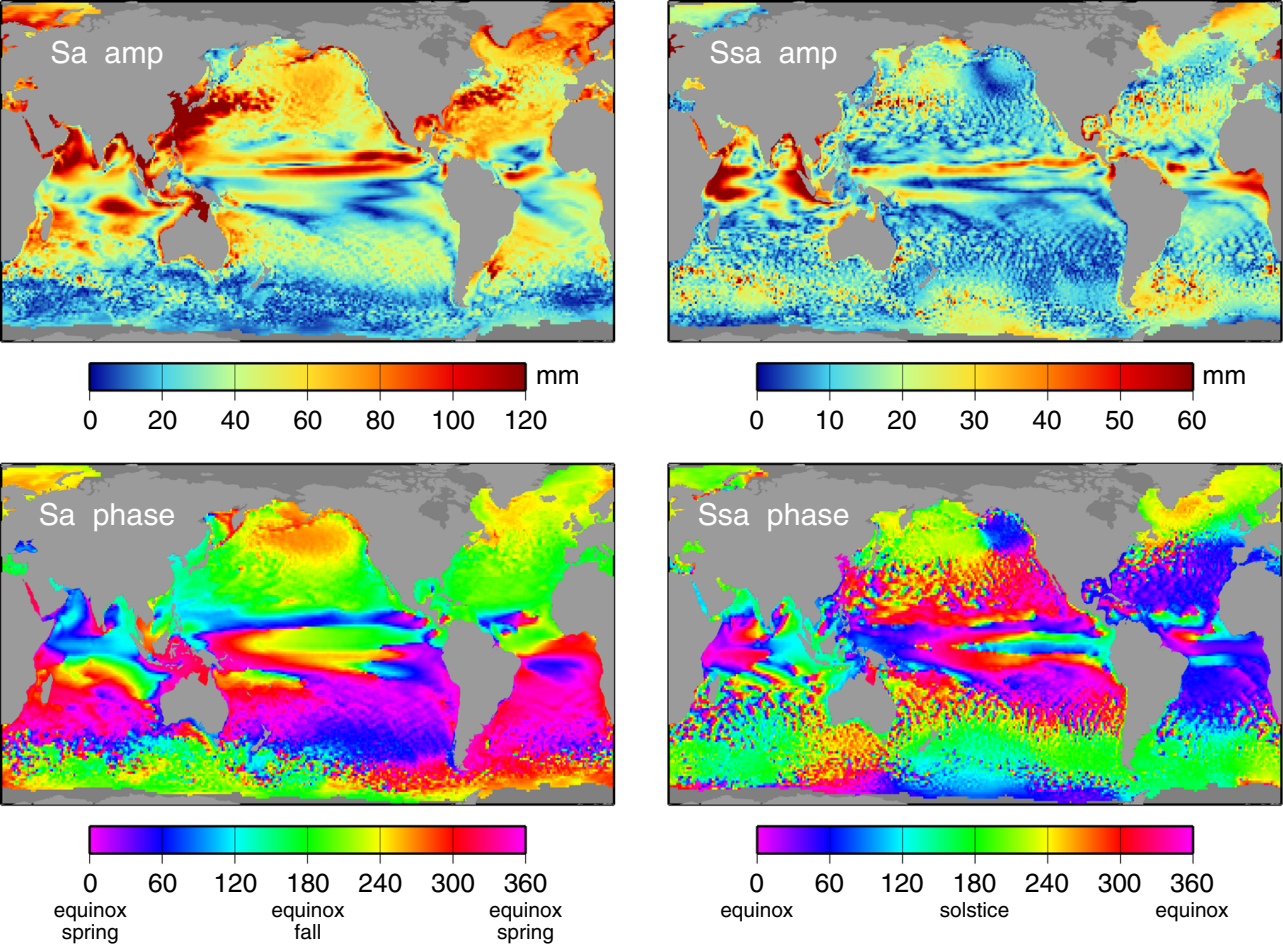
Amplitudes (top panels) and phase lags (bottom panels) of the annual (Sa) and semiannual (Ssa) sea-level oscillations in *relative* sea level. Results are based on data of Fig. 1, but with tides and IB restored and corrected for vertical land motion and geocenter motion

The final charts of annual Sa and semiannual Ssa amplitudes and phases of *relative* sea level are shown in Fig. 8. These can be compared with similar charts in Fig. 1 of absolute sea level, presented in a form generally displayed in the published literature, i.e., without tidal or IB contributions. The amplitudes of both Sa and Ssa in Fig. 8 are generally larger, especially in the higher latitudes where the IB and tidal contributions become especially pronounced. The North Atlantic, including the northwest European Shelf, and the East China Sea show markedly larger amplitudes. For various applications that require the full sea-level signal, and not just the dynamic part—e.g., analysis and prediction of coastal flooding—these higher amplitudes are of some importance.

The land motion component of relative sea level, at both the annual period and especially the semiannual period, is small and therefore less important to the final result than the large IB term. The annual amplitude of vertical deformation is actually smaller than the standard errors depicted in Fig. 3 at 505 of our 544 tide gauges. (Recall that those standard errors are based on the year-to-year variability in observed Sa.) The amplitude of vertical deformation exceeds the Sa signal itself at only one station: Rarotonga in the Cook Islands. Thus, in most applications, the land motion component could well be neglected, as in practice it typically is, even in comparison studies like ours (e.g., Vinogradov and Ponte 2010; Ruiz Etcheverry et al. 2015), Nonetheless, it is reassuring that the altimeter data are now sufficiently precise that the small land motion term from GRACE can still reduce the RMS values of Table 1 by 1–2%. At least for the MEaSUREs data, the geocenter adjustment can reduce the RMS values another 2%.

Assuming the limited GRACE spatial resolution is not missing large, but localized, land motion, we may ask what are the main contributors to the altimeter and tide-gauge differences. The differences in results between MEaSUREs and DUACS altimetry suggest that a large part owes to differences in objective mapping algorithms, in the quantity of altimetry (DUACS employed data from more missions), and/or in altimeter corrections (e.g., for tides, mean sea surface, wet troposphere delay). Yet there are clearly differences with tide gauges common to both altimeter products. Unless measurement error or outliers (e.g., land contamination in the altimeter footprint) are involved, the cause(s) of altimeter and tide-gauge differences must be that different ocean signals are being observed, usually because of the different spatial resolutions of the two systems. It is enlightning to examine cases of especially large differences, for which the causes may sometimes be more readily discerned. Two examples follow.

### West coast of North America

**Fig. 9:**
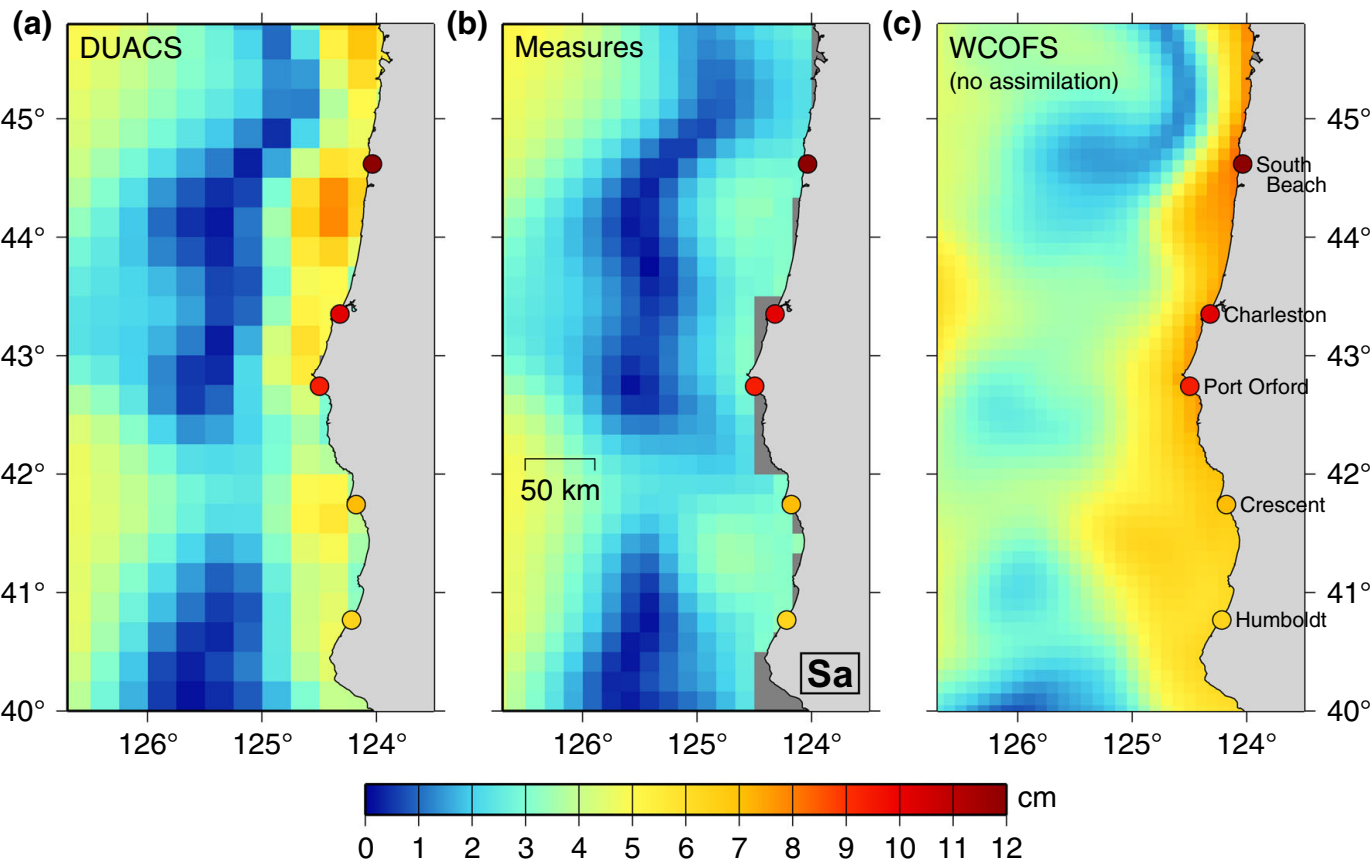
Amplitude (cm) of the annual (Sa) sea-level oscillation off the west coast of Oregon and California, for **a** DUACS altimetry, **b** MEaSUREs altimetry, and **c** the West Coast Ocean Forecast System (Kurapov et al. 2017), run without data assimilation. **a** is a zoom view from Fig. 8. Color dots are Sa amplitudes at tide gauges. **c** Amplitudes were kindly computed and provided by Alexander Kurapov

Examination of the differences between the 544 tide gauges and the DUACS altimeter data reveals that, of the twelve gauges with the largest differences, five are located on the west coast of North America. This problematic location was also noted by Vinogradov and Ponte (2010), and is generally understood to be caused by the intense California Current and strong offshore upwelling along the coast (Strub et al. 1987). Figure 9 compares the Sa amplitudes of DUACS and MEaSURES altimetry (the former a magnified view from Fig. 8) with the high-resolution (2 km) West Coast Ocean Forecast System (WCOFS) described by Kurapov et al. (2017). The model was run without data assimilation of any kind, so it is not biased in favor of either altimeter or tide-gauge data. The model indicates enhanced annual amplitudes along a narrow band, between 30 and 100 km wide, off the Oregon-California coast. Both altimeter products do show higher amplitudes closer to the coast, but they are still too small, thus resulting in large altimeter/tide-gauge differences.

The WCOFS model itself fails to capture the large amplitudes at the northern tide gauges, especially for South Beach (Oregon) where the Sa amplitude is 12 cm. This may stem from inadequacies in the model. But note that this gauge is actually located inside Yaquina Bay, which is fed by the Yaquina River, and we suspect that the large annual amplitude owes in part to river discharge, which peaks in mid-winter, in-phase with the annual phase of the WCOFS model. Wave setup (next section) in winter could also be a contributor. The Charleston gauge, located within the mouth of Coos Bay, may also be affected by river discharge. Port Orford, directly open to the sea, is likely less affected by discharge.

It is possible that present-day satellite altimetry could capture more of the narrow band of high amplitudes along the coast by fine-tuning of gridding algorithms and near-coastal correlation scales. Also, some recent altimeters like Sentinel-3A use so-called Delay Doppler technology, which also allows improved mapping near coasts (Fenoglio et al. 2021). However, mapping the transition from river to ocean, which is possibly necessary at South Beach, will require a swath altimeter, of the sort expected to appear in the near future (Durand et al. 2010; Morrow et al. 2019).

### Minamitori Island

Of all test tide gauges located in the open ocean, the largest Sa discrepancy with altimetry occurs at Minamitori Island (called Marcus Island on most older maps). The island is a very small ($1.5~\hbox {km}^2$) atoll located about 2000 km southeast of Tokyo, at 24$^{\circ }$ $17.2^{'}$ N, 153$^{\circ }$ $58.8^{'}$ E. At this location, altimetry indicates an annual amplitude and phase lag of 4.7 cm, 225$^{\circ }$(DUACS) and 4.6 cm, 225$^{\circ }$(MEaSUREs), respectively. The island tide gauge (for the period 1997–2013) gives $16.1 \pm 1.4~\hbox {cm}$, $281^{\circ }\pm 5^{\circ }$. The discrepancy with altimetry is pronounced in both amplitude and phase. Note that the tide gauge is reported to be a pressure recorder. Limited documentation implies that atmospheric pressure has been removed from the measurements; a coherence calculation suggests otherwise, but this is not definitive because the pressure variability at this location is small. In any event, the annual IB amplitude is 2.5 cm, too small (and with the wrong phase) to explain the discrepancy between tide gauge and altimeter. A GPS station on the island (station MCIL) indicates annual vertical motion of no more than a few mm, so land motion is also not involved.

In this section, we lay out evidence for attributing this large discrepancy to wind-generated waves—and more specifically to an annual cycle in wave setup. The small island, surrounded by a coral reef, has no harbor or natural shelter, so the tide gauge is directly exposed to effects of wave breaking on the reef. Wave setup occurs when the gradient of momentum flux associated with wave breaking is balanced by a slope in mean sea level (Longuet-Higgins and Stewart 1962), thus raising sea level between the reef and the tide gauge (Pugh and Woodworth 2014). While wave setup is typically studied on short time scales associated with periods of very high waves, where setup-driven sea level extremes can be of order 1 m (e.g., Hoeke et al. 2013; Vetter et al. 2010), it is also appreciated that setup can lead to smaller, yet significant, long period (seasonal and interannual) changes in mean sea level (Melet et al. 2018; Pugh and Woodworth 2014).

In any given coastal environment the interaction among bottom topography, the incident wave field, and sea level is complex, and wave setup depends on fine-scale details (e.g., of reef topography). For example, wave setup observed at the Tristan da Cunha (South Atlantic) tide gauge changed markedly once a jetty was extended at the small harbor (Woodworth 2020). Such detailed information is not generally available for modeling and understanding setup without special field campaigns. Alternatively, empirical parameterizations can be developed (Stockdon et al. 2006), not uncommonly with wave setup taken as some fraction—typically from 10 to 30%—of offshore significant wave height, where the constant of proportionality is dependent on many factors.

**Fig. 10:**
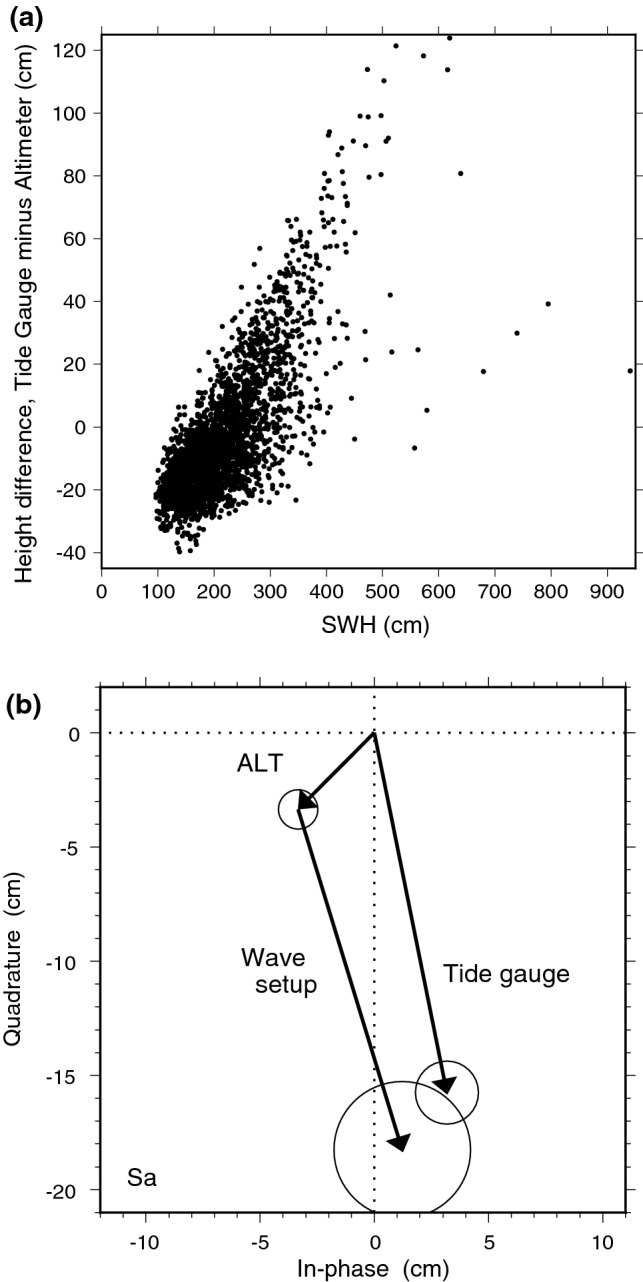
Wave setup at Minamitori Island. **a** Scatter diagram of daily sea level observations at the island tide gauge, minus corresponding measurements from altimetry, plotted as a function of significant wave height (SWH). There is a clear correlation between sea level differences and SWH (correlation coefficient 0.76), which we attribute to wave setup on the island. (b) Components of the Minamitori annual cycle Sa, allowing for wave setup calculated as a fraction (27%) of SWH, which is seen to close the discrepancy between tide gauge and altimeter estimates. A factor of 0.27 is in keeping with other, more detailed, investigations of wave setup (e.g., Vetter et al. 2010)

**Fig. 11:**
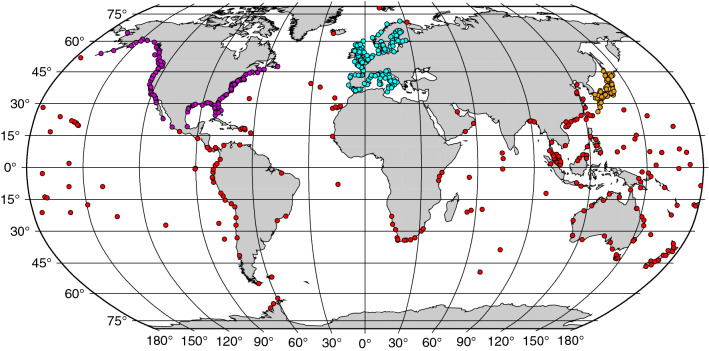
Locations of tide gauges used for comparison with altimetry. Source of these data was GESLA-2 (Woodworth et al. 2017). Different colors mark clusters of stations used in subregions of Tables 1 and 2

To determine if a similar parameterization is viable at Minamitori, we have compiled daily mean sea levels, subtracted corresponding DUACS altimetry to remove the low-frequency variability, and compared with daily offshore significant wave height (SWH) from ERA5 reanalysis. For the time period 2010–2017, the results are shown in Fig. 10a. The correlation is visually striking (Pearson coefficient is 0.76). A number of high-SWH points in the right half of the diagram, fall off the main high-correlation cluster, and they evidently cannot be described by this wave-setup model, but these points are relatively few. (The largest SWH, over 9 m according to ERA5, occurred on 2015 Oct 5, when Typhoon Choi-wan passed directly over the island.) The main cluster of points in the figure follows approximately a straight line with slope 0.31. Orthogonal regression, when applied to all the data, yields a slightly smaller slope of 0.27. The formal regression uncertainty is $\pm 0.03$, but given the uncertainty in the model, this should be at least doubled.

According to ERA5 reanalysis, the offshore SWH over the period 1993–2020 has an annual cycle amplitude of $58 \pm 4~\hbox {cm}$ and phase of $287^{\circ }\pm 4^{\circ }$. Scaling by 0.27 gives a wave setup amplitude of 15.7 cm. This contribution to the annual cycle should be added to that observed offshore by altimetry, to be compared with the tide gauge measurement. The Sa vector diagram for these three components is shown in Fig. 10b. The agreement is seen to be quite good.

Notwithstanding this good agreement, a far more in-depth analysis, outside the scope of this paper, is warranted, because wave setup must depend on wave direction relative to the tide gauge’s location on the island, on wave period, on wind waves versus swell versus infragravity waves, on reef geometry and other factors. Nevertheless, the evidence of Fig. 10—both the correlation and the Sa vector closure—is sufficient to establish that wave setup is playing a large role in the measured annual sea-level cycle at the tide gauge. It also raises the question as to how much wave setup is similarly affecting other tide-gauge measurements. While the altimeter/tide-gauge discrepancy was greater at Minamitori than at any other island in our test dataset, there are many coral atolls in the global sea level network known to be susceptible to high-wave flooding (Hoeke et al. 2013), and there are coastal tide gauges located on unsheltered shores exposed to the surf or in harbors with known setup (e.g., Thompson and Hamon 1980; Dodet et al. 2019). One suspects that wave setup, even if substantially smaller than in Fig. 10, may still be contributing some part to altimeter and tide-gauge differences. The question deserves careful study, since it bears on many other sea level (and altimeter) studies, including measurement validation.

## Concluding remarks

Satellite altimetry is a technology developed strictly for monitoring the open oceans, but it is now being pushed ever more toward near-coastal waters (Vignudelli et al. 2011). Our “ground truth” tide-gauge data for the annual and semiannual cycles in relative sea level suggest that altimetry is capable of providing comparably useful estimates along most coasts: Sa signals exceeding 6 cm in RMS yield altimeter-minus-gauge differences of less than 2 cm RMS for DUACS altimetry, and somewhat larger with MEaSUREs altimetry. It is reassuring that, with few exceptions (see Tables 1 and 2), the agreement between altimetry and the test tide gauges improves with each adjustment of the altimetry. This includes the adjustment for annual variations in vertical land motion, which are here based on a high-resolution inversion of satellite gravimetry, even though annual deformation is generally only a few mm (Fig. 7).

The use of satellite gravimetry necessarily limits the spatial resolution of estimated vertical land motion. Recent work with InSAR data has emphasized the sometimes surprising complexity and fine scales of land motion, at least for linear rates (e.g., Buzzanga et al. 2020; Blackwell et al. 2020). Annual land motion has been less studied at short scales, but it is known to occur where hydrological loading is large and localized, such as from snow loading (e.g., Silverii et al. 2020). Thus, work toward improving the spatial resolution is an obvious route to improved relative sea levels, especially if it can be established that GRACE spatial resolution is missing important localized deformation.

So too will on-going improvements in coastal altimetry, along the lines described by Vignudelli et al. (2019) and Fenoglio et al. (2021). Updated versions of DUACS and MEaSUREs altimetry will eventually incorporate some of these improvements. Such work will likely reduce altimeter and tide-gauge discrepancies in places like the US west coast (Fig. 9). Reconciling shorter scale differences, however, such as very localized effects of, say, river discharge, will likely require a time series of swath altimeter measurements; the sea level community eagerly anticipates swath data in the near future (Morrow et al. 2019). Wave setup acts at even smaller scales, which are challenging to observe remotely in any detail, but its effects evidently appear in altimeter comparisons (Fig. 10) and merit further attention.

## Figures and Tables

**Fig. 12:**
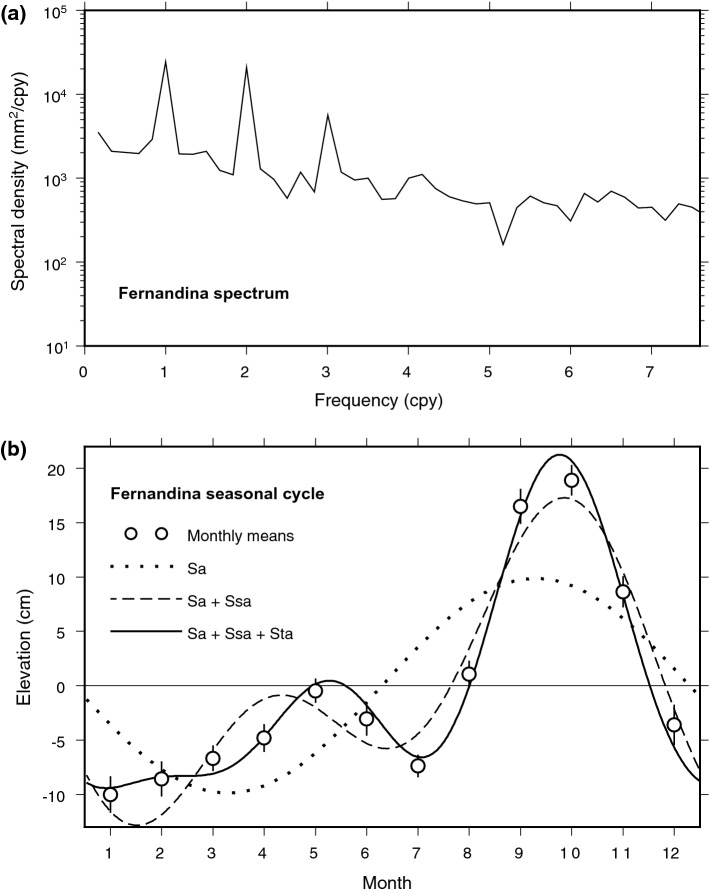
**a** Sea level spectrum at Fernandina Beach, Florida, showing 3 clear peaks at 1, 2, and 3 cycles per year. The spectrum was computed from 13 Welsh segments, each of 6 years of daily mean sea levels without gaps, covering the period 1898–2019. **b** Seasonal cycle at Fernandina based on data from 1985 to 2019. Open circles are monthly means. Curves are summations of the three seasonal harmonics, where the amplitudes of Sa, Ssa, and Sta are 9.9, 8.0, and 4.1 cm, respectively

## Data Availability

DUACS altimeter data are available from the Copernicus Marine Service https://marine.copernicus.eu. MEaSUREs altimeter data are available from the Jet Propulsion Laboratory data archive https://podaac.jpl.nasa.gov/MEaSUREs-SSH. The ECMWF ERA5 pressure data are available from the Copernicus Climate Change Service https://cds.climate.copernicus.eu. Tide-gauge data are available from https://www.gesla.org/. Additional datasets generated during this work are available from the corresponding author on reasonable request.

## Appendix A Argument and phase conventions

In discussions of the seasonal cycle, it is not uncommon to express phases as a day number or a month number since the beginning of the calendar year. Reckoning or sampling by months has little to recommend it, other than perhaps tradition. As Cartwright (1983) emphasized, “the irregular lengths of calendar months from 28 to 31 days and their nonsensical time sequence make for nonuniform sampling of averages and irregular intervals between them.” Days are less objectionable, but still have nonuniform number in a year. Adopting a phase origin at January 1 does bring concordance with common civil time, but the beginning of the Gregorian calendar year corresponds to nothing physical. More importantly, for any quantity that is truly seasonal and thus tied to insolation and thereby to the equinox, a time origin of January 1 introduces unnecessary noise in phase estimation. This is manifested most obviously by the changing dates of the equinox. This phase noise may be of little consequence in many applications, but as we study climate change and attempt to identify possibly small but important changes in phase, this unnecessary calendar jitter should be avoided. Stine and Huybers (2012) have made similar points in the context of studying changes in the seasonal cycle of air temperature; in particular, see their Figure A2(d).

There is a preferable alternative. For at least a century (Doodson 1928), and probably much earlier (e.g., Harris 1895, Table 1) the tidal community has parameterized the observed annual cycle in sea level in terms of the sun’s mean ecliptic longitude. The annual oscillation—still routinely labeled the Sa constituent even though the argument for the Sa astronomical potential is different (see below)—is written as $A \cos [ \Theta (t) - G ]$, with amplitude *A* and phase lag *G*, and where the argument $\Theta (t)$ equals simply *h*, the mean longitude of the sun relative to the vernal equinox. The period of oscillation is then exactly one tropical year. A simple relationship can be used to evaluate *h* at any time:

2 $$\begin{aligned} h = 280.466 + 36000.7698 \, T \end{aligned}$$

in degrees, where *T* is time in Julian centuries (36 525 d) since noon, January 1, 2000. Higher-order expansions, taken up to $T^4$, may be found in Meeus (1998), but these terms are unnecessary for times of interest here. Use of the sun’s mean longitude provides a uniform time argument for the observed seasonal cycle, without calendar noise and appropriately tied to the equinox and to the physical origin of seasonality.

Note that *h* is the mean longitude, not the true longitude, of the sun. The true longitude is a nonlinear function of time and its use in the argument of Sa would result in a nonharmonic function. The mean longitude, in contrast, advances uniformly in time (assuming we neglect the possible high-order terms in (2)). As such, passage of *h* through 0$^{\circ }$ does not correspond to the exact time of the spring equinox, which in any event is generally defined as the moment when the *apparent* longitude of the sun is zero (Seidelmann 1992); the latter includes high-frequency nutational motions which would also be undesirable in the argument for Sa.

The preceding paragraphs refer to the observed annual cycle in sea level, which is predominantly the ocean’s response to insolation and the associated climate effects. The gravitational tidal constituent Sa, which is usually only a very small component of the observed annual cycle (Sect. 4), must be handled differently. As noted above, the Sa constituent arises as a modulation of the sun’s permanent tide induced by the annually varying distance between earth and sun. Because the earth’s orbital eccentricity is small, the Sa astronomical potential is small, as is apparent from the amplitudes in Fig. 5. The potential is maximum at perihelion, minimum at aphelion. Its argument $\Theta (t)$ should thus be taken as the mean longitude of the sun relative to perihelion, so that the argument is properly zero at time of maximum potential. In terms of Doodson’s standard variables, $\Theta = h - p_s$, where $p_s$ is the mean longitude of perihelion, reckoned like *h* relative to the vernal equinox. (With this argument, the Doodson number for Sa is then 056.554.) This is the argument that appears in the tidal potential catalogs of Doodson (1921) and Cartwright and Tayler (1971). The period of the gravitational tide is one anomalistic year rather than one tropical year.

This leads to a common source of confusion whether the argument of the annual cycle is taken as *h* or $(h-p_s)$. In principle, the choice should depend on whether one is referring to the observed sea-level oscillation, induced primarily by climate, or to the gravitational oscillation forced by astronomical motions. Most national agencies responsible for tidal analysis and prediction adopt $\Theta = h$. Schureman (1940) explicitly mentions that Sa should be considered “a meteorological rather than an astronomical constituent,” and US authorities continue to use $\Theta =h$ in their tidal tables.^1^ Likewise, Doodson and Warburg (1941, Table 7.1), following Doodson (1928), use the same argument.

However, there are exceptions. The popular software package of Foreman (1977), and therefore also that of Pawlowicz et al. (2002) which is based on Foreman’s package, adopts the gravitational argument $\Theta = (h - p_s)$. Estimated phases from those software packages therefore differ from those published by (say) NOAA by the mean value of $p_s$. (The current value of $p_s$ is approximately 283.3$^{\circ }$; in 1960 it was 1$^{\circ }$ smaller.)

Thus, when using compilations of harmonic constants, one must take care to understand the convention used for the argument of Sa. In addition to the constants routinely released by NOAA, the harmonic constants produced by Ponchaut et al. (2001) and those stored as part of the Global Undersea Pressure (GLOUP) program similarly adopted $\Theta = h$. The old collection of over 4000 sets of tidal constants compiled under the auspices of the International Hydrographic Bureau (Ritchie 1980)—now officially withdrawn although copies survive in the hands of many tide researchers—follows the same convention. The even older table of Sa and Ssa constants compiled for about 200 worldwide stations by Harris (1907) also used $\Theta = h$, as did other collections of constants published in that era (e.g., Darwin 1889). On the other hand, the recent compilation of harmonic constants by Piccioni et al. (2019), who analyzed times series from the GESLA database (Woodworth et al. 2017), adopted the gravitational argument $\Theta = (h-p_s)$. In practical terms, in light of the slow speed of $p_s$—it completes one revolution in 209 centuries—either convention is acceptable so long as consistency between tidal analysis and tidal prediction is maintained. Over many centuries, however, the use of the gravitational argument would be inappropriate because, tied to perihelion, it would fail to move with the precession of the equinoxes.

No similar confusions arise for the semiannual Ssa oscillation. The argument is always taken as 2*h* (except, of course, for those authors who take phases relative to January 1). The problem potentially arises again for Sta, because the gravitational potential includes $p_s$ in its argument (Cartwright and Tayler 1971), but Sta is small and is rarely considered in any study. In our work above, we took its argument as 3*h*.

For readers who need to convert our equinox-relative phase lags to a convention that uses day-of-year phase, add approximately 80$^{\circ }$ to our values for Sa, and approximately 160$^{\circ }$ for Ssa.

## Appendix B Test tide gauges

A set of test tide gauges, from which seasonal harmonics have been estimated from hourly measurements, forms a critical part of this study, as they allow us to assess how accurately the altimeter height data can be converted to relative sea levels. This appendix briefly describes the selected test tide gauges.

The source for these test data was the GESLA-2 database of tide-gauge measurements compiled by Woodworth et al. (2017). The full database contains data from 1276 tide gauges, although about 200 of these are duplicates (but not always covering identical time spans). Some stations are inappropriate for this study because, for example, they are located far up rivers (e.g., Philadelphia and Washington in the United States) or in water nearly isolated from the ocean (e.g., gauges in the Seto Inland Sea in Japan). Some of the inappropriate stations could be eliminated by automated means, but many required “hand editing.” All selected time series were required to have at least 9 years of nearly complete measurements; this ensured a degree of statistical robustness in the estimated seasonal constants (Tsimplis and Woodworth (1994) set a limit of at least 5 years). In an attempt to maintain (rough) consistency with the time span of altimeter data, only data after 1985 were used. A few stations were eliminated in locations where there was little hope of obtaining altimetry throughout the year (e.g., Churchill and Prudhoe Bay), owing to ice cover. We were left with data from 544 tide gauges. Locations are shown in Fig. 11.

At each station a full tidal analysis was computed, generally for about 110 constituents, including for present purposes Sa, Ssa, and Sta. For the 544 stations, the median amplitude of Sa was found to be 7.1 cm; of Ssa 2.2 cm; and of Sta 0.7 cm.

The largest Sa amplitudes occur at several stations in the Ganges Delta of Bangladesh, most notably the tide gauge at Charchanga in the Meghna Estuary where the amplitude reaches 50 cm (a few stations farther upstream have even larger amplitudes, but were removed). The very large seasonal oscillations in that area have been known for some time (e.g., Pattullo et al. 1955). The next largest oscillation, among the 544 tide gauges, is at Booby Island, Australia (just to the west of Torres Strait in the upper Gulf of Carpentaria), where the mean Sa amplitude is 29 cm; the large amplitudes in that region are evident in Fig. 1. Stations with the largest Ssa amplitudes are found in the Gulf of Mexico, with amplitudes 6–8 cm. The largest Sta amplitudes are two stations on the Atlantic coast of Florida, St. Augustine and Fernandina Beach, both slightly over 4 cm.

A detailed depiction of the mean seasonal cycle at Fernandina is shown in Fig. 12b. Peak sea level occurs in October and a much lower secondary peak in May. The slow rise from January through April is one reason why all three harmonics are needed to fully describe the seasonal cycle and to match the Fernandina monthly means (open circles). But the seasonal cycle at Fernandina, with an Sta harmonic with amplitude 4.1 cm, is not typical; 90% of examined tide gauges have Sta amplitudes less than 1.5 cm.

## Appendix C MEaSUREs gridded altimetry

The MEaSUREs gridded altimeter time series is briefly described in this appendix, with attention primarily on the gridding methodology. Further information is given by Zlotnicki et al. (2019). Processing of the Topex and Jason altimetry is described by Beckley et al. (2019, 2017), with the choice of reference frame and other properties relevant to this work noted briefly in Sect. 7.

The gridding procedure was simple kriging (Cressie and Wikle 2011). A covariance function for each location over the global oceans was constructed as an analytical function given by

$$\begin{aligned} C_{ij}= & {} \left\langle h(x_i,y_i,t_i) \cdot h(x_j,y_j,t_j) \right\rangle \\= & {} V_0 b \exp (-a r_{ij} - ({\text {d}}t_{ij}/L_t)^2) \end{aligned}$$

where $\left\langle \cdot \right\rangle$ denotes an expected value operator, implemented as an average over pairs separated by the same ${\text {d}}x, {\text {d}}y, {\text {d}}t$, and *h*(*x*, *y*, *t*) is the sea surface height at horizontal position *x*, *y* and time *t*. The decay scale $L_t$ was set to 10 days for latitudes up to 5$^{\circ }$, 15 days for latitudes above 10$^{\circ }$, and linearly in between. Other terms defining $C_{ij}$ are:

$$\begin{aligned} a= & {} 3.3369,\\ b= & {} 1 + r + r^2/6 - r^3/6,\\ r= & {} \surd \{(({\text {d}}x-C_x {\text {d}}t)/L_x)^2 + (({\text {d}}y-C_y {\text {d}}t)/L_y)^2\} \end{aligned}$$

where ${\text {d}}x=x_i-x_j$, and similarly for ${\text {d}}y$ and ${\text {d}}t$. The factor $V_0$ is the variance of *h* observed from Topex and Jason along-track data over the interval 1993–2014. $L_x$ and $L_y$ are length scales in the zonal and meridional directions determined from historical altimetry by Gregg Jacobs (pers. communication, 2013). $C_x$ and $C_y$ are drift velocity components determined directly from the altimetry by an iterative method. In a first step, $C_x, C_y$ were set to zero and the complete altimeter dataset was used to generate grids sampled every (1/6)$^{\circ }$ and 5 days. The drift velocities were then determined by analyzing successive grids, shifting them in position to maximize cross-correlations. The final step reran the whole gridding computation using the $C_x$ and $C_y$ maps thus determined.

Given the above covariance function, the estimation of $h(P),\,\, P=(x,y,t)$ is the linear combination

$$\begin{aligned} h(P)= \sum _i h(Q_i)\, w_i(P,Q_i) \end{aligned}$$

where the vector *w* of weights $w_i$ was estimated by least squares as

$$\begin{aligned} \left( \begin{array}{c} w \\ \mu \end{array} \right) = \left( \begin{array}{cc} D &{} 1\\ 1 &{} 0 \end{array} \right) ^{-1} \; \left( \begin{array}{c} C \\ 1 \end{array} \right) \end{aligned}$$

where *C* is the set of $C_{ij}$ between the position of grid node *i* and each of the surrounding data points *j*; $D=C^{\prime }+E$ is the sum of the matrix of $C^{\prime }_{ik}$ between any two data points *i* and *k* and the expected error covariance $E_{ik}$ between those two positions. The $E_{ik}$ was taken as diagonal except when two data points were on the same groundtrack, where a bias error was allowed and solved for. This track bias was assumed to be $(1\,\hbox {cm})^2$ for the Topex and Jason satellites, and $(3\,\hbox {cm})^2$ for the ERS-1, ERS-2, Envisat, and AltiKa satellites. These weights are important in the context of Sect. 7.

## References

[CR1] Agnew DC, Farrell WR (1978). Self-consistent equilibrium ocean tides. Geophys J R astr Soc.

[CR2] Altamimi Z, Rebischung P, Métivier L, Collilieux X (2016). ITRF2014: a new release of the International Terrestrial Reference Frame modeling nonlinear station motions. J Geophys Res Solid Earth.

[CR3] Amiruddin AM, Haigh ID, Tsimplis MN, Calafat FM, Dangendorf S (2015). The seasonal cycle and variability of sea level in the South China Sea. J Geophys Res Oceans.

[CR4] Barbosa SM, Donner RV (2016). Long-term changes in the seasonality of Baltic sea level. Tellus A.

[CR5] Beckley BD, Callahan PS, Hancock DW, Mitchum GT, Ray RD (2017). On the ‘cal-mode’ correction to TOPEX satellite altimetry and its effect on the global mean sea level time series. J Geophys Res Oceans.

[CR6] Beckley BD, et al (2019) Integrated multi-mission ocean altimeter data for climate research: TOPEX/Poseidon, Jason-1, 2, & 3: User’s handbook. 10.5067/ALTTS-TJ142

[CR7] Blackwell E, Shirzaei M, Ojha C, Werth S (2020). Tracking California’s sinking coast from space: implications for relative sea-level rise. Sci Adv.

[CR8] Buzzanga B, Bekaert DPS, Hamlington BD, Sangha SS (2020) Toward sustained monitoring of subsidence at the coast using InSAR and GPS: an application in Hampton Roads. Virginia. Geophys Res Lett 47:e2020GL090013. 10.1029/2020GL090013

[CR9] Calafat FM, Wahl T, Lindsten F, Williams J, Frajka-Williams E (2018). Coherent modulation of the sea-level annual cycle in the United States by Atlantic Rossby waves. Nat Commun.

[CR10] Carrère L, Lyard F (2003). Modeling the barotropic response of the global ocean to atmospheric wind and pressure forcing—comparisons with observations. Geophys Res Lett.

[CR11] Carrère L, Faugère Y, Ablain M (2016). Major improvement of altimetry sea level estimations using pressure-derived corrections based on ERA-Interim atmospheric reanalysis. Ocean Sci.

[CR12] Carton JA (1983). The variation with frequency of the long-period tides. J Geophys Res.

[CR13] Cartwright DE (1983). On the smoothing of climatological time series, with application to sea-level at Newlyn. Geophys J R astr Soc.

[CR14] Cartwright DE, Tayler RJ (1971). New computations of the tide-generating potential. Geophys J R astr Soc.

[CR15] Chanard K, Fleitout L, Calais E, Barbot S, Avouac JP (2018). Constraints of transient viscoelastic rheology of the asthenosphere from seasonal deformation. Geophys Res Lett.

[CR16] Chanard K, Fleitout L, Calais E, Rebischung P, Avouac JP (2018). Toward a global horizontal and vertical elastic load deformation model derived from GRACE and GNSS station position time series. J Geophys Res Solid Earth.

[CR17] Chen G, Qian C, Zhang C (2012). New insights into annual and semiannual cycles of sea level pressures. Mon Wea Rev.

[CR18] Collilieux X, Altamimi Z, Ray J, van Dam T, Wu X (2009). Effect of the satellite laser ranging network distribution on geocenter motion estimation. J Geophys Res.

[CR19] Couhert A, Cerri L, Legeais JF, Ablain M, Zelensky NP, Haines BJ, Lemoine FG, Bertiger WI, Desai SD, Otten M (2015). Towards the 1 mm/y stability of the radial orbit error at regional scales. Adv Space Res.

[CR20] Cressie N, Wikle CK (2011). Statistics for spatio-temporal data.

[CR21] Darwin GH (1889). Second series of results of the harmonic analysis of tidal observations. Proc R Soc.

[CR22] Davis JL, Elósegui P, Mitrovica JX, Tamisiea ME (2004). Climate-driven deformation of the solid Earth from GRACE and GPS. Geophys Res Lett.

[CR23] Desai SD, Bertiger W, Haines BJ (2014). Self-consistent treatment of tidal variations in the geocenter for precise orbit determination. J Geod.

[CR24] Desai SD, Wahr JM, Beckley BD (2015). Revisiting the pole tide for and from satellite altimetry. J Geod.

[CR25] Dodet G, Melet A, Ardhuin F, Bertin X, Idier D, Almar R (2019). The contribution of wind-generated waves to coastal sea-level changes. Surv Geophys.

[CR26] Dong D, Fang P, Bock Y, Cheng MK, Miyazaki S (2002). Anatomy of apparent seasonal variations from GPS-derived site position time series. J Geophys Res.

[CR27] Doodson AT (1921). The harmonic development of the tide-generating potential. Proc R Soc.

[CR28] Doodson AT (1928). The analysis of tidal observations. Philos Trans R Soc.

[CR29] Doodson AT, Warburg HD (1941). Admiralty manual of tides.

[CR30] Durand M, Fu LL, Lettenmaier DP, Alsdorf DE, Rodriguez E, Esteban-Fernandez D (2010). The Surface Water and Ocean Topography mission: observing terrestrial surface water and oceanic submesoscale eddies. Proc IEEE.

[CR31] Egbert GD, Ray RD (2003). Deviation of long-period tides from equilibrium: kinematics and geostrophy. J Phys Oceanogr.

[CR32] Ekman M, Stigebrandt A (1990). Secular change of the seasonal variation in sea level and of the pole tide in the Baltic Sea. J Geophys Res.

[CR33] Farrell WE (1972). Deformation of the earth by surface loads. Rev Geophys Space Phys.

[CR34] Feng X, Tsimplis MN, Marcos M, Calafat FM, Zheng J, Jorda G, Cipollini P (2015). Spatial and temporal variations of the seasonal cycle in the northwest Pacific. J Geophys Res Oceans.

[CR35] Fenoglio L, Dinardo S, Uebbing B, Buchhaupt C, Gärtner M, Staneva J, Becker M, Klos A, Kusche J (2021). Advances in NE-Atlantic coastal sea level change monitoring by Delay Doppler altimetry. Adv Space Res.

[CR36] Foreman MGG (1977) Manual for tidal heights analysis and prediction. Pacific Marine Sci. Rep. 77–10, Institute of Ocean Sciences, Sidney, B.C

[CR37] Gill AE, Niiler PP (1973). The theory of the seasonal variability in the ocean. Deep Sea Res.

[CR38] Harris RA (1895) Manual of tides, Part III. Some connections between harmonic and nonharmonic quantities, including applications to the reduction and prediction of tides. In: Report of the Superintendent of the U. S. Coast & geodetic survey: progress of the work, fiscal year ending (June 1894) Part II. Government Printing Office, Washington, pp 125–262

[CR39] Harris RA (1907) Manual of tides, Part V. Currents, shallow-water tides, meteorological tides, and miscellaneous matters. In: Report of the Superintendent of the Coast & Geodetic survey: progress of the work [Fiscal year ending June 1907], Government Printing Office, Washington, pp 231–546

[CR40] Hersbach H (2020). The ERA5 global reanalysis. Quart J R Met Soc.

[CR41] Hoeke RK, McInnes KL, Kruger JC, McNaught RJ, Junter JR, Smithers SG (2013). Widespread inundation of Pacific islands triggered by distant-source wind-waves. Glob Planet Change.

[CR42] Jacobs GA, Born GH, Parke ME, Allen PC (1992). The global structure of the annual and semiannual sea surface height variability from Geosat altimeter data. J Geophys Res.

[CR43] Knudsen P (1994). Global low harmonic degree models of the seasonal variability and residual ocean tides from Topex/Poseidon altimeter data. J Geophys Res.

[CR44] Kurapov A, Erofeeva SY, Myers E (2017). Coastal sea level variability in the US West Coast Ocean Forecast System (WCOFS). Ocean Dyn.

[CR45] Kusche J, Schrama EJO (2005). Surface mass redistribution inversion from global GPS deformation and GRACE gravity data. J Geophys Res.

[CR46] Lambeck K (1980). The Earth’s variable rotation: geophysical causes and consequences.

[CR47] Lau N, Borsa AA, Becker TW (2020) Present-day crustal vertical velocity field for the contiguous United States. J Geophys Res Solid Earth 125:e2020JB020066. 10.1029/2020JB020066

[CR48] Lemoine FG (2010). Towards development of a consistent orbit series for TOPEX, Jason-1, and Jason-2. Adv Space Res.

[CR49] Li W, van Dam T, Li Z, Shen Y (2016). Annual variation detected by GPS, GRACE and loading models. Stud Geophys Geod.

[CR50] Longuet-Higgins MS, Stewart RW (1962). Radiation stress and mass transport in gravity waves, with application to surf-beats. J Fluid Mech.

[CR51] Loomis BD, Richey AS, Arendt AA, Appana R, Deweese YJC, Forman BA, Kumar SV, Sabaka TJ, Shean DE (2019). Water storage trends in high mountain Asia. Front Earth Sci.

[CR52] Lyard F, Allain D, Cancet M, Carrère L, Picot N (2021) FES2014 global ocean tides atlas: design and performances. Ocean Sci 17 (in press)

[CR53] Meehl GA (1991). A reexamination of the mechanism of the semiannual oscillation in the southern hemisphere. J Clim.

[CR54] Meeus J (1998). Astronomical algorithms.

[CR55] Melet A, Meyssignac B, Almar R, Le Cozannet G (2018). Under-estimated wave contribution to coastal sea-level rise. Nat Clim Change.

[CR56] Morrow R (2019). Global observations of fine-scale ocean surface topography with the Surface Water and Ocean Topography (SWOT) mission. Front Mar Sci.

[CR57] Munk WH, Cartwright DE (1966). Tidal spectroscopy and prediction. Philos Trans R Soc.

[CR58] Nerem RS, Schrama EJ, Koblinsky CJ, Beckley BD (1994). A preliminary evaluation of ocean topography from the TOPEX/POSEIDON mission. J Geophys Res.

[CR59] Passaro M, Cipollini P, Vignudelli S, Quartly GD, Snaith HM (2014). ALES: a multi-mission adaptive subwaveform retracker for coastal and open ocean altimetry. Remote Sens Environ.

[CR60] Pattullo J, Munk W, Revelle R, Strong E (1955). The seasonal oscillation in sea level. J Mar Res.

[CR61] Pawlowicz R, Beardsley B, Lentz S (2002). Classical tidal harmonic analysis including error estimates in matlab using t\_tide. Comput Geosci.

[CR62] Piccioni G, Dettmering D, Bosch W, Seitz F (2019). TICON: Tidal constants based on GESLA sea-level records from globally located tide gauges. Geosci Data J.

[CR63] Plag HP, Tsimplis MN (1999). Temporal variability of the seasonal sea level cycle in the North Sea and Baltic Sea in relation to climate variability. Glob Planet Change.

[CR64] Ponchaut F, Lyard F, Le Provost C (2001). An analysis of the tidal signal in the WOCE sea level dataset. J Atmos Oceanic Tech.

[CR65] Ponte RM, Chaudhuri AH, Vinogradov SV (2015). Long-period tides in an atmospherically driven, stratified ocean. J Phys Oceanogr.

[CR66] Pugh DT, Woodworth PL (2014). Sea level science: understanding tides, Surges, Tsunamis and mean sea-level changes.

[CR67] Pujol MI, Faugère Y, Tauburet G, Dupuy S, Pelloquin C, Ablain M, Picot N (2016). DUACS DT2014: the new multi-mission altimeter data set reprocessed over 20 years. Ocean Sci.

[CR68] Ritchie GS, Sears M, Merriman D (1980). Some aspects of the history of oceanography as seen through the publications of the International Hydrographic Bureau. Oceanography: the past.

[CR69] Rudenko S, Otten M, Visser P, Scharroo R, Schöne T, Esselborn S (2012). New improved orbit solutions for the ERS-1 and ERS-2 satellites. Adv Space Res.

[CR70] Ruiz Etcheverry LA, Saraceno M, Piola AR, Valladeau G, Möller OO (2015). A comparison of the annual cycle of sea level in coastal areas from gridded satellite altimetry and tide gauges. Cont Shelf Res.

[CR71] Sagiya T, Miyazaki S, Tada T (2000). Continuous GPS array and present-day crustal deformation of Japan. Pure Appl Geophys.

[CR72] Schureman P (1940) Manual of harmonic analysis and prediction of tides. Spec. Publ. 98, U.S. Coast & Geodetic Survey, Washington, p 317

[CR73] Seidelmann PK (1992). Explanatory supplement to the astronomical Almanac.

[CR74] Silverii F, Montgomery-Brown EK, Borsa AA, Barbour AJ (2020) Hydrologically induced deformation in Long Valley Caldera and adjacent Sierra Nevada. J Geophys Res Solid Earth 125:e2020JB019495. 10.1029/2020JB019495

[CR75] Stine AR, Huybers P (2012). Changes in the seasonal cycle of temperature and atmospheric circulation. J Clim.

[CR76] Stockdon HF, Holman RA, Howd PA, Sallenger AH (2006). Empirical parameterization of setup, swash, and runup. Coast Eng.

[CR77] Strub PT, Allen JS, Huyer A, Beardsley RC (1987). Seasonal cycles of currents, temperature, winds, and sea level over the Northeast Pacific continental shelf. J Geophys Res.

[CR78] Taburet G, Sanchez-Roman A, Ballarotta M, Pujol MI, Legeais JF, Fournier F, Faugère Y, Dibarboure G (2019). DUACS DT2018: 25 years of reprocessed sea level altimetry products. Ocean Sci.

[CR79] Tesmer V, Steigenberger P, van Dam T, Mayer-Gürr T (2011). Vertical deformations from homogeneously processed GRACE and global GPS long-term series. J Geod.

[CR80] Thompson RORY, Hamon BV (1980). Wave setup of harbor water levels. J Geophys Res.

[CR81] Tregoning P, Lambeck K, Ramillien G (2008). GRACE estimates of sea surface height anomalies in the Gulf of Carpentaria, Australia. Earth Planet Sci Lett.

[CR82] Tsimplis MN, Woodworth PL (1994). The global distribution of the seasonal sea level cycle calculated from coastal tide gauge data. J Geophys Res.

[CR83] van Dam T, Wahr J, Lavallée D (2007). A comparison of annual vertical crustal displacements from GPS and GRACE over Europe. J Geophys Res.

[CR84] van Loon H (1967). Modes of atmospheric variability over the Southern Ocean. J Atmos Sci.

[CR85] Vetter O, Becker JM, Merrifield MA, Pequignet AC, Aucan J, Boc SJ, Pollock CE (2010). Wave setup over a Pacific Island fringing reef. J Geophys Res.

[CR86] Vignudelli S, Kosianoy AG, Cipollini P, Benveniste J (2011). Coastal altimetry.

[CR87] Vignudelli S, Birol F, Benveniste J, Fu LL, Picot N, Raynal M, Roinard H (2019). Satellite altimetry measurements of sea level in the coastal zone. Surv Geophys.

[CR88] Vinogradov SV, Ponte RM (2010). Annual cycle in coastal sea level from tide gauges and altimetry. J Geophys Res.

[CR89] Vinogradov SV, Ponte RM, Heimbach P, Wunsch C (2008). The mean seasonal cycle in sea level estimated from a data-constrained general circulation model. J Geophys Res.

[CR90] Wahl T, Calafat FM, Luther ME (2014). Rapid changes in the seasonal sea level cycle along the US Gulf coast from the late 20th century. Geophys Res Lett.

[CR91] Walland D, Simmonds I (1999). Baroclinicity, meridional temperature gradients, and the southern semiannual oscillation. J Clim.

[CR92] Woodworth PL (2020). Wave setup at Tristan da Cunha. Afr J Mar Sci.

[CR93] Woodworth PL, Hunter JR, Marcos M, Caldwell P, Menéndez M, Haigh I (2017). Towards a global higher-frequency sea level dataset. Geosci Data J.

[CR94] Wu X, Ray J, van Dam T (2012). Geocenter motion and its geodetic and geophysical implications. J Geodyn.

[CR95] Zlotnicki V, Qu Z, Willis J, Ray R, Hausman J (2019) JPL MEASURES gridded sea surface height anomalies, Vers. 1812, PO.DAAC, Pasadena. 10.5067/SLREF-CDRV2

